# Unveiling poly(rC)-binding protein 2 as the target protein for curcusone C against prostate cancer: mechanism validation through click chemistry-activity based proteomics profiling approach

**DOI:** 10.1186/s12885-023-11467-0

**Published:** 2023-10-09

**Authors:** Lan Huang, Buqing Ma, Chong Zhang, Jiaqi Shi, Rui Shen, Erci Zhang, Chenlei Lian, Cuifang Wang, Jieqing Liu

**Affiliations:** 1 School of medicine, Huaqiao University, Quanzhou, 362021 China; 2 Quanzhou Normal University, Quanzhou, 362000 China

**Keywords:** Prostate cancer, CC-ABPP, Target protein, PCBP2, Mechanism validation

## Abstract

**Background:**

Prostate cancer is a disease that seriously troubles men. However, there are some inevitable limitations in interventional therapy for prostate cancer patients at present, most of which are caused by low selectivity and high toxic side effects due to unclear drug targets. In this study, we identified the target protein of Curcusone C with anti-prostate cancer potential activity and verified its target and mechanism of action.

**Methods:**

Click chemistry-activity based proteomics profiling (CC-ABPP) method was used to find target protein of Curcusone C against prostate cancer. Competitive CC-ABPP, drug affinity responsive target stability (DARTS) and surface plasmon resonance (SPR) methods were used to verifying the target protein. Moreover, potential mechanism was validated by western blot in vitro and by *he*matoxylin-eosin (HE) staining, detection of apoptosis in tumor tissue (TUNEL), and immunohistochemical (IHC) in vivo.

**Results:**

We found that poly(rC)-binding protein 2 (PCBP2) was the target protein of Curcusone C. In addition, Curcusone C might disrupt the Bax/Bcl-2 balance in PC-3 cells by inhibiting the expression of the target protein PCBP2, thereby inducing mitochondrial damage and activation of the mitochondrial apoptosis pathway, and ultimately inducing apoptosis of prostate cancer cells.

**Conclusions:**

Curcusone C is a potential compound with anti-prostate cancer activity, and this effect occurs by targeting the PCBP2 protein, which in turn may affect the TGF/Smad signaling pathway and Bax/Bcl-2 balance. Our results laid a material and theoretical foundation for Curcusone C, to be widely used in anti-prostate cancer.

**Supplementary Information:**

The online version contains supplementary material available at 10.1186/s12885-023-11467-0.

## Background

Cancer is a disease which unregulated growth and proliferation of cells invade normal tissue cells in a variety of ways [1]. Prostate cancer is a common form of cancer, according to the European Cancer Results [2]. Prostate cancer deeply affects men’s health, and its prevalence is still increasing in the future [3, 4]. We urgently need to focus on the occurrence, development and treatment in prostate cancer. However, androgen deprivation therapy and early chemotherapy are commonly applied to prostate cancer patients now [5]. Such intervention methods often cause a certain physical and mental burden on patients, and the side effects are large. Therefore, it is necessary to develop new therapies with selective treatment and minor side effect.

In recent years, various studies have shown that natural products provided new, efficient, and safe treatment ideas for cancer patients. There is also a growing awareness that natural products can not only treat cancer but also prevent it [6]. Diterpenoids are widely present in natural plants and have shown potential in curing cancer [7]. Curcusones, natural diterpenoids, were first obtained from the roots of *Jatropha curcas* L. in 1986 [8]. In our previous studies, we obtained a large number of Curcusones from the roots of Jatropha carcass L. in the early stage, and found that these compounds had significant anti-tumor activity [9]. Excitingly, Curcusone C, a member of the Curcusones family of compounds, has been shown to have promising antitumor activity. Wang et al. found that the interaction between Curcusone C and telomere repeat binding factor 2 (TRF2) blocked the interaction between shelterin, a telomere protective protein complex, and telomere DNA, thereby activating the DNA damage response and generating cancer cell cycle arrest as well as apoptosis [10]; Zhang’s group found Curcusone C had significant effects on anti-proliferation and apoptosis on human endometrial cancer cell line HEC-1 A, and the further mechanism studies showed Curcusone C induced mitochondrial pathway induced apoptosis by inducing cytochrome C release and Bax activation [11]. Although the antitumor activity and mechanism of Curcusones C of compounds are well understood, the identification of specific targets is indispensable, which contributes to more reliable mechanistic studies and greatly restricts application and development of Curcusone C.

Target identification is a critical process and major challenge in the development of natural drugs, not only because the interaction between natural products and intracellular target proteins is the basis of their pharmacological activities, but also because target identification can provide effective information for determining the toxic and side effects of natural products [12, 13]. In recent years, CC-ABPP has shown great potential in the process of drug development and target identification [14]. The application of CC-ABPP technology is likely to provide great help in finding the antitumor target of Curcusone C.

Thus, in this study, Cursusone C was the template for the structural modifications and 15 derivatives were obtained. Based on their bioassay results of anti-prostate cancer, their structure-active relationship was constructed. Furthermore, we identified the anti-prostate cancer targets of Curcusone C using CC-ABPP and verified the target protein using western blot, competitive CC-ABPP, DARTS and SPR methods. In addition, we validated the mechanism of Curcusone C against prostate cancer through signal pathway and in vivo experimental analyses. Our results highlight the feasibility of Curcusone C in treating prostate cancer, paving the way of the development and application of novel anti-prostate cancer drugs.

## Methods

### Antibodies

GAPDH (bs-2188R), PARP (bs-2138R), p-Smad7 (bs-20024R), Smad7 (bs-0566R), p-Smad2 (bs-2224R), Smad2 (bs-0718R), Bax (bs-0127R) and Bcl-2 (bs-0032R) antibodies were obtained from Bioss (Beijing, China). Cleaved-Caspase 3 (AF7022), Caspase 3 (AF6311) and Cleaved-PARP (AF7023) antibodies were purchased from Affinity Biosciences LTD (OH, USA). FHL3 (11028-2-AP), TGF-β1 (21898-1-AP), PCBP2 (15070-1-AP) were obtained from ProteinTech (IL, USA).

### Cell line and cell culture

Human prostate carcinoma PC-3 cells and human proximal tubular HK-2 cells purchased from Chinese National Collection of Authenticated Cell Cultures were cultured in RPMI 1640 medium containing FBS (10%, Gibco, CA, USA) and Penicillin-Streptomycin (1%, Gibco, CA, USA). The cells were cultured at a concentration of 5% CO_2_ and a temperature of 37 ℃, and the medium was changed every two days.

### Curcusone C

Curcusone C was isolated and extracted from the roots of *J. curcas*. The *J. curcas* were collected with permission in Shuangbai County Chuxiong Prefecture Yunnan province, in July 2016. The collection of the *J. curcas* used in our study complied with local or national guidelines, and were identified by Dr Jun He (Kunming Institute of Botany, Chinese Academy of Sciences). A voucher specimen (No. HQU 20,160,701) has been deposited at School of Biomedical Sciences, Huaqiao 131 University, Quanzhou, P. R. China.

### Synthesis of curcusone C derivatives

Derivative C-1. Using 1.5 mL CH_2_Cl_2_ dissolved Curcusone C (15.2 mg, 0.048 mmol), and DMF of 2 drops and SO_2_Cl_2_ (7.0 µL, 0.096 mmol) were added. Following the reflux reaction by TLC, and the reaction was stopped about 1.5 h later. After drying the solvent, diluting with water, and extracting with ethyl acetate, and the organic layer was washed with saturated salt. Drying with anhydrous Na_2_SO_4_, decompression and concentration, derivative C-1 (light yellow solid, 9.3 mg, 57.8%) was purified by PTLC (PE: EtOAc = 5:1).

Derivative C-2. Using 1.5 mL THF dissolved Curcusone C (33.7 mg, 0.108 mmol), tert-butyl hypochlorite (18.3 µL, 0.162 mmol) was added in ice bath. The reaction was gradually raised from 0 °C to room temperature. TLC followed the reaction for about 10 h, and the reaction was stopped and diluted with water. Derivative C-2 (yellow oil, 26.6 mg, 67.6%) was obtained after decompression concentration and purification by PTLC (PE: EtOAc = 4:1).

Derivative C-3. Using 1 mL DMF dissolved Curcusone C (14.8 mg, 0.047 mmol), adding iodine elemental (71.2 mg, 0.281 mmol). The reaction proceeded at room temperature. The reaction was followed by TLC and was stopped after 15 min. After adding sodium thiosulfate solution into the reaction solution, the organic layer was washed with saturated salt. After drying with anhydrous Na_2_SO_4_, decompression and concentration, derivative C-3 (light yellow solid, 3.9 mg, 18.0%) was purified by PTLC (PE: EtOAc = 4:1).

Derivative C-13. Curcusone C (25.1 mg, 0.080 mmol) was dissolved in 1 mL THF, and tert-butyl hypochlorite (27.3 µL, 0.240 mmol) was added in ice bath. The reaction was gradually increased from 0 °C to room temperature, followed by TLC, and stopped about 24 h later. After diluting with water, extracting with ethyl acetate for 3 times, using saturated salt washing organic layer, drying with anhydrous Na_2_SO_4_, and condensing under pressure by PTLC (PE: EtOAc = 4: 1), derivative C-13 (yellow oily, 27.9 mg, 87.0%) was purified.

Derivative C-4. Using 1.5 mL DMSO dissolved derivative C-13. Then, KH_2_PO_4_ and catalytic KI (5% equiv) were added, and heated to 80 °C for about 20 h. Quenched with water after the reaction was complete, and extracted with ethyl acetate for 3 times. Then, combining with organic layer, washing with saturated salt, and drying by anhydrous Na2SO4. Derivative C-4 (28.0 mg, 52.2%, after two steps) was purified by PTLC (CH_2_Cl_2_: MeOH = 60:1) after decompression concentration.

Derivative C-5. Derivative C-13 (14.2 mg, 0.036 mmol) was dissolved in 1 mL acetone, followed by the addition of potassium acetate (14.2 mg, 0.140 mmol) and catalytic amount of KI (5% equiv). The reaction was carried out at room temperature, followed by TLC tracking reaction. After about 48 h, the reaction was complete and stopped. The combined organic layer was dried with anhydrous Na_2_SO_4_. After decompression concentration, derivative C-5 (yellow solid, 8.4 mg, 55.9%) was purified by PTLC (PE: EtOAc = 2:1).

Derivative C-6. Derivative C-13 was dissolved in 1.5 mL acetone followed by reflux reaction with potassium sorbate (58.2 mg, 0.376 mmol) and a catalytic amount of KI (5% equiv). TLC tracked reaction, and the reaction was complete about 30 h later. The reaction was stopped, and the acetone was dried under pressure, diluted with water, and ethyl acetate extraction for 3 times. The combined organic layer was dried with anhydrous Na_2_SO_4_, condensed under pressure and purified by PTLC (CH_2_Cl_2_: MeOH = 80:1) to obtain derivative C-6 (6.9 mg, 15.1% after two steps).

Derivative C-7. Derivative C-13 (20.7 mg, 0.052 mmol) was dissolved in 1 mL acetone, followed by the addition of KI (13.2 mg, 0.080 mmol), and the reaction was stopped at room temperature for 20 h. The acetone was dried under pressure, dichloromethane was dissolved, the insoluble matter was filtered out, and the crude product was obtained by drying. The crude product was dissolved in 1.6 mL DMSO: H_2_O = 3:5. Then, cuprous oxide (14.2 mg, 0.099 mmol) was added to the solvent, heated to 50 °C. The reaction was stopped for about 8 h, the insoluble matter was filtered out, diluted with water, extracted with ethyl acetate for 3 times, and the combined organic layer was dried with anhydrous Na_2_SO_4_. After decompression, the concentration was processed by PTLC (PE: EtOAc = 2: 1). Derivative C-7 (yellow solid, 7.1 mg, 36.0%) was purified.

Derivative C-8. Derivative C-13 (14.2 mg, 0.036 mmol) was dissolved in 1 mL acetone, followed by the addition of potassium acetate (14.2 mg, 0.140 mmol) and catalytic amount of KI (5% equiv). The reaction was carried out at room temperature, followed by TLC tracking reaction. After about 48 h, the reaction was complete and stopped. The combined organic layer was dried with anhydrous Na_2_SO_4_. After decompression and concentration, derivative C-8 (yellow solid, 8.4 mg, 55.9%) was purified by PTLC (PE: EtOAc = 2:1).

Derivative C-9. Derivative C-13 (20.2 mg, 0.051 mmol) was dissolved in 1.5 mL acetone, followed by the addition of mono-methyl malonate potassium salt (33.0 mg, 0.211 mmol) and catalytic amount of KI (5% equiV). The reflux reaction was followed by TLC tracking reaction. After about 48 h, the reaction was complete. The combined organic layer was dried with anhydrous Na_2_SO_4_ and purified by PTLC (PE: EtOAc = 2:1) after decompression and concentration, derivative C-9 (yellow oily, 13.6 mg, 56.5%) was obtained.

Derivative C-10. Derivative C-2 (14.5 mg, 0.040 mmol) was dissolved in 1 mL DMF, followed by potassium acetate (6.0 mg, 0.061 mmol), reacted at room temperature. The reaction was followed by TLC, and stopped for about 12 h. Then, the reaction was diluted with water, extracted with ethyl acetate for 3 times, and washed with saturated salt for the combined organic layer. Derivative C-10 (yellow oily, 6.3 mg, 40.8%) was purified by PTLC (PE: EtOAc = 3:1) after anhydrous Na_2_SO_4_ drying and decompression concentration.

Derivative C-11. Derivative C-2 (16.8 mg, 0.046 mmol) was dissolved in 1 mL DMF, diethylamine (9.5 µL, 0.092 mmol) and K_2_CO_3_ (19.9 mg, 0.144 mmol) were added to react at room temperature, followed by TLC, and the reaction was stopped for about 12 h, diluted with water. Derivative C-11 (yellow oily, 5.6 mg, 30.4%) was obtained after decompression concentration and purification by PTLC (PE: EtOAc = 3:1).

Derivative C-12. Derivative C-13 (26.0 mg, 0.0651 mmol) was dissolved in 1.5 mL acetone, followed by the addition of sodium p-nitrophenol (32.1 mg, 0.163 mmol) and catalytic amount of KI (5% equiV). The reaction was performed at room temperature, followed by TLC follow-up reaction, and the reaction was complete and stopped about 24 h later. The combined organic layer was dried with anhydrous Na_2_SO_4_. After decompression and concentration, derivative C-12 was purified by PTLC (CH_2_Cl_2_: MeOH = 70:1) to obtain a brown-yellow solid, 11.1 mg, 28.3%.

Derivative C-14. Derivative C-13 (28.9 mg, 0.072 mmol) was dissolved in 1.5 mL acetone, followed by the addition of potassium indolebutyrate (37.2 mg, 0.144 mmol) and catalytic amount of KI (5% equiv). The reflux reaction was followed by TLC tracking reaction. After about 48 h, the reaction was complete and stopped. The combined organic layer was dried with anhydrous Na_2_SO_4_. After decompression and concentration, derivative C-14 was purified by PTLC (PE: EtOAc = 2:1) to obtain an oily brown-yellow color, 10.1 mg, 24.6%.

Derivative C-15. Curcusone C (19.2 mg, 0.062 mmol), hydroxylamine hydrochloride (7.2 mg, 0.104 mmol) and potassium acetate (12.1 mg, 0.123 mmol) were dissolved in 1.5 mL methanol. The reflux reaction was followed by TLC, and the reaction was stopped after about 4 h. The organic layer was washed with saturated salt, dried with anhydrous Na_2_SO_4_, concentrated under reduced pressure, and purified by PTLC (PE: EtOAc = 2:1) to obtain derivative C-15 (yellow solid, 14.3 mg, 67.4%).

Positive probe. Hexylic acid (16.5 µL, 0.150 mmol) was dissolved in 1.5 mL CH_2_Cl_2_, and SO_2_Cl_2_ (163 µL, 2.250 mmol) was added for reflux reaction. The reaction was stopped about 8 h later. The CH_2_Cl_2_ and SO_2_Cl_2_ were removed under pressure to obtain the crude product of hexylic chloride. Added 1 mL CH_2_Cl_2_ to dissolve, drop by drop into the sample solution of CH_2_Cl_2_ with Cur C (15.2 mg, 0.048 mmol), Et_3_N (20.3 µL, 0.146 mmol) and DMAP (catalytic volume, 5%). The reflux reaction was stopped after about 12 h. The organic layer was dried with anhydrous Na_2_SO_4_. After decompression, PTLC (PE: EtOAc = 5:1) was concentrated to obtain positive probe (yellow oily, 12.7 mg, 64.2%).

Negative probe. The positive probe (22.1 mg, 0.054 mmol) was dissolved in 1.5 mL methanol, followed by the addition of hydroxylamine hydrochloride (5.8 mg, 0.083 mmol) and potassium acetate (9.0 mg, 0.091 mmol). The reflux reaction was followed by TLC, and stopped about 4 h later. Methanol was distilled under pressure, diluted with water, extracted with ethyl acetate. Then, the organic layer was washed with water and dried with anhydrous Na_2_SO_4_. PTLC (PE: EtOAc = 3:1) was concentrated under pressure to obtain negative probe (yellow solid, 10.5 mg, 43.9%).

The instrument used in this part are AVANCE III-400 NMR instrument (Bruck, Switzerland), AVANCE III-500 NMR instrument (Bruck, Switzerland) and QSTAR Plusar I mass spectrometer (Thermo, USA).

### Cytotoxic activity assay

CCK-8 assay was used to evaluate the proliferation and cytotoxicity of Curcusone C and its derivatives. Cells of 100 µL with a concentration of 5000 cells/mL to 8000 cells/mL were inoculated in 96-well plates for culturing 24 h, and the blank well was added with equal volume medium. After cell adhesion, 10 µL Curcusone C solution or its derivatives were added to each well of the experimental groups except the blank well to the final concentration of drug was 1 mM, 2 mM, 5 mM, 10 mM and 20 mM respectively, and 10 µL DMSO (drug solvent) was added to the control well. The liquid was discarded 48 h later, and 100 µL medium and 10 µL CCK-8 reagent (Beyotime, Shanghai, China) were added to each well. After incubation for 0.5 to 1 h, the absorbance valued at 450 nm was measured and the cell inhibition rate or survival rate was calculated. Cell inhibition rate= [(negative control well OD blank well OD)/ (experimental well OD blank well OD)] × 100%.

### CC-ABPP

2 mL positive probe storage solution and 2 mL negative probe solution were added to the PC-3 containing medium to achieve a final concentration of 20 µM. The above liquid was incubated at 37 ℃ for 2 h. Then, cells were collected, proteins were prepared by 30% power ultrasound and the protein concentration was regulated by PBS at 1 mg/mL to 2 mg/mL. The samples were grouped and added into 5 mM biotin azide compound of 11.3 µL, 50 mM TCEP (Aladdin, Shanghai, China) of 11.3 µL, 1.7 mM TBTA (Aladdin) of 34 µL and 50 mM CuSO_4_·5H_2_O (Macklin, Shanghai, China) of 11.3 µL. The protein was incubated at room temperature for 1 h to precipitate. After centrifugation, pre-cooled methanol was added for washing three times and the protein was dissolved. 50 µL streptavidin magnetic beads were added to each group of samples, and the magnetic beads were precipitated and washed three times. The supernatant of samples was discarded and 30 µl PBS (Gibco, CA, USA) and 30 µL 5X protein loading buffer was added to the samples for boiling at 95 ℃ for 10 min. SDS-PAGE electrophoresis was performed and the gel was stained with silver or coomassie bright blue.

### Western blot

The culture medium containing PC-3 cells was discarded, lysis buffer was added, and the cells were collected with a scraper. The sample is then transferred to a centrifuge tube and the cells are fully lysed. Centrifuge the tube at 12,000 g and 4 ℃ for 10 min. After taking the supernatant, mix with the protein: protein loading buffer (v/v) = 5:1 ratio, and boil at 95 ℃ for 10 min to fully denaturate the protein.

BCA protein concentration assay kit (Beyotime, China) was used to quantify the protein of experimental samples. Equal amounts of protein (30 µg) were loaded onto 7.5-12.5% SDS-PAGE gel. After adding the sample, the target protein was separated at 140 V constant pressure and was transferred under the condition of 250 mA constant current and the highest voltage. The transferred PVDF membrane (Thermo Fisher Scientific, MA, USA) was sealed in 5% skim milk (BIOFRox, Germany) for 2 h. The membrane was incubated in the antibody diluent of the corresponding protein at 4 ℃ overnight in a shaker and washed with TBST (Gibco, USA) for three times. Then it was placed in secondary antibody diluent for incubating 1.5 h at 4 ℃ in a shaker and washed again for three times. Finally, appropriate amount of ECL luminescent solution was taken to infiltrate PVDF membrane for development, and the strip was exposed and recorded.

### DARTS

PC-3 cells protein was extracted, and the protein concentration was adjusted to 5 mg/mL and was packed into 99 µL/tube. Then, 10 mM and 5 mM Curcusone C solution of 1 µL was added to protein mixture of 99 µL and gently shaken at room temperature for 1 h. After the compounds were completely incubated, the samples were divided into one part every 20 µL, and 2 µL pronase solution with a certain concentration was added. The control group was added with 2 µL TNC buffer and incubated at room temperature for 30 min to ensure the same digestion time in each group. Digestion was terminated with 20X protease inhibitor complex and incubated on ice for 10 min. Finally, 6 µL 5X protein loading buffer was added to each sample and boiled at 100 ℃ for 10 min to fully denaturate the protein. The samples were centrifuged at 1100 g and 4 ℃ for 1 min and were performed western blot.

### SPR

The PCBP2 protein was first immobilized on the CM5 microarray using an immunoassay, and then the analyte was run using a multi-cycle method. Briefly, small molecules were diluted to different concentrations with Running Buffer, and injected into channel Fc1-Fc2 at a flow rate of 30 µl/min. The association period was 60 s, and then dissociated for 90 s. The association and dissociation processed in the Running Buffer.

### Animal experiment

PC-3 cells in logarithmic growth phase were collected and the total number of cells required was calculated. Cells were re-suspended in a suitable volume of PBS and added to matrigel (PBS: matrigel = 1:1(v/v)) at a concentration of 5 × 10^6^ cells/mL for subcutaneous inoculation on the right side of Balb/C nude mice. On day 0, 0.1 mL 5 × 10^6^ cells/mL cell suspension (PBS: matrigel = 1:1(v/v)) was subcutaneously inoculated on the right neck and back of each nude mouse except the mice of normal group, to establish PC-3 subcutaneously transplanted tumor model. On day 8 after cell inoculation, the tumor grew to an average volume of 140.3 mm^3^. The groups were randomly divided into normal group (Normal), control group (Control), methotrexate group (MTX 4.8 mg/kg), low dose group (C 5 mg/kg), medium dose group (C 10 mg/kg) and high dose group (C 20 mg/kg). Except for 3 mice in the normal group, 6 mice in the other groups were administered twice a week via tail vein (BIW). After 2 weeks of dosing, the dose was changed to once a day (QD). After 2 days of QD administration, tail vein injection was changed to intraperitoneal administration, and QD administration was continued for 5 days. Pharmacodynamic activity was evaluated based on tumor inhibition rate (TGITV) and tolerance was evaluated based on animal weight change and death. At the end of the experiment, all the animals were bled under isoflurane anesthesia and the serum was extracted. The heart, liver, spleen, lung and kidney of each nude mouse were collected and fixed in 4% paraformaldehyde or quick-frozen in liquid nitrogen and stored at -80 ℃ for tissue protein extraction.

All animal experiments were complied with the National Research Council’s Guide for the Care and Use of Laboratory Animals guidelines, and the program was approved by the Animal Experiment Ethical Review Committee of Huaqiao University (NO. A2020017). The study is reported in accordance with ARRIVE guidelines.

### HE staining

Tissue samples were embedded, sectioned, dewaxed, stained, dehydrated and transparent, and sealed with neutral gum. After drying in a ventilated place, observing and recording under a microscope, the sections were stored at room temperature.

### Detection of apoptosis in tumor tissue (TUNEL)

Mouse tumor tissues were fixed with 4% PFA and washed with PBS, and TUNEL staining was performed with TUNEL apoptosis detection kit according to manufacturer’s instructions. Cell nucleus were stained with DAPI, slides were rinsed and imaged with fluorescence microscopy, and finally apoptosis was analyzed with image analyzer.

### Immunohistochemical (IHC)

Paraffin sections underwent successively dewaxing to water, antigen repair, blocking of endogenous peroxidase, serum blocking, primary antibody incubation, secondary antibody reaction, DAB color development, counterstaining of nuclei, dehydration and sealing, image acquisition and analysis, and microscopic examination.

### Statistical analysis

The experimental data were shown by mean ± standard error of mean (SEM). The experimental data were calculated and analyzed by GraphPad Prism and SPSS, and the final results were obtained from three independent experimental results.

## Results

### Evaluation of curcusone C against prostate cancer in vitro

We primarily evaluated the toxicity of Curcusone C to human prostate carcinoma PC-3 cells and human proximal tubular HK-2 cells respectively in vitro by CCK-8 method (Table 1). Results showed the IC_50_ of Curcusone C to PC-3 was about 3.24 µM, which was nearly three times that of the clinical drug methotrexate. In addition, the IC_50_ of Curcusone C to HK-2 was greater than 20 µM. However, the results of toxic activity of methotrexate on normal cells showed that the IC_50_ of methotrexate was about 3.21 µM, indicating that the toxic activity of Curcusone C on normal cells was much lower than methotrexate. These results indicated that Curcusone C had significant toxic activity to PC-3 but little toxic activity to normal cells.

**Table 1:** Cytotoxic activity of Curcusone C and its derivatives.

Compounds	PC-3 (IC_50_ in µM)	HK-2 (IC_50_ in µM)
Methotrexate	0.89 ± 0.22	3.21 ± 0.68
Curcusone C	3.24 ± 0.16	> 20
C-1	3.74 ± 0.40	-
C-2	6.40 ± 0.38	-
C-3	10.34 ± 0.58	-
C-4	2.35 ± 0.37	-
C-5	2.05 ± 0.96	-
C-6	1.81 ± 0.18	-
C-7	16.33 ± 3.25	-
C-8	9.49 ± 0.17	-
C-9	5.97 ± 1.16	-
C-10	> 20	-
C-11	> 20	-
C-12	> 20	-
C-13	2.96 ± 0.37	-
C-14	4.10 ± 0.23	-
C-15	>>20	-
Positive probes	7.34 ± 0.20	-
Negative probes	>>20	-

### Probe synthesis and activity evaluation

The identification of target proteins by CC-ABPP is based on the synthesis of molecular probes, and the synthetic probes should retain the structure and anti-prostate cancer activity of Curcusone C.

**Fig. 1:**
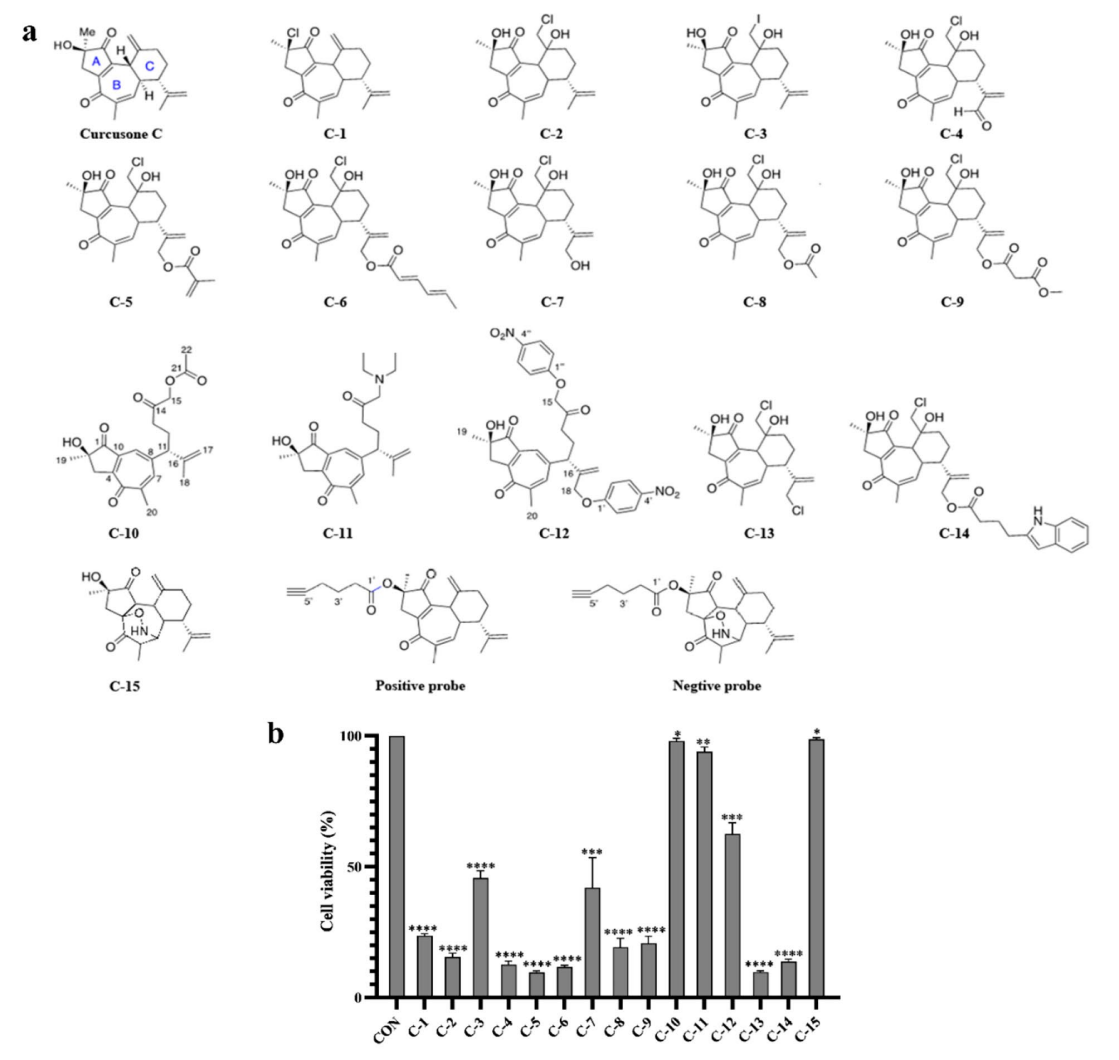
Curcusone C derivatives and their anti-prostate cancer activity. **a**, structures of Curcusone C and its derivatives; **b**, cell survival rate of sixteen derivatives incubated with PC-3 cell. Cells in control group were treated with DMSO. Compared with the 0 µM control group, *p* > 0.05, *p < 0.05, **p < 0.01, ***p < 0.001, ****p < 0.0001

The structure of Curcusone C was shown in Fig. 1a. In this study, we left the B ring unchanged and modified the other structural parts, especially the C ring. In short, for A ring, chlorine group was introduced on 2-hydroxyl group; the C ring was modified by two terminal double bonds and allyl position. The modified Curcusone C derivative was shown in Fig. 1a.

Based on the structure-activity relationship study result that 2-OH had high reactivity and little effect on the anti-prostate cancer activity, molecular probes were synthesized to introduced an alkynyl group into 2-OH for identifying the target of Curcusone C by CC-ABPP combined with mass spectrometry, and the structure of probes were shown as Fig. 1a, and the structure information of derivatives were shown in Supp. Figure 16 and Supp. Figure 17.

Then, we also evaluated the toxicities of positive probe and negative probe to PC-3 cells in vitro by CCK-8 method. According to the analysis of the cytotoxic activities to PC-3 (Table 1), the inhibitory rate of 20 µM negative probe on PC-3 was only 20%, and its IC_50_ was far greater than 20 µM. Interestingly, the IC_50_ of the modified positive probe was 7.34 ± 0.20 µM, indicating that the positive probe not only retained the chemical framework of Curcusone C, but also retained toxic activity to PC-3 cells to a certain extent.

**Fig. 2:**
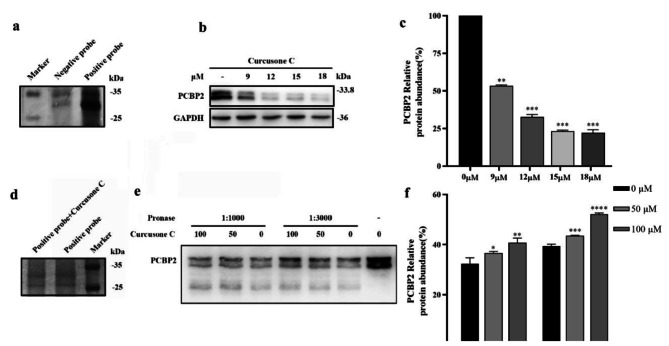
Identification and verification of Curcusone C target protein. **a**, the results of SDS-PAGE Gel silver staining; **b**, western blot results of PCBP2 in PC-3 cells under different concentrations of Curcusone C; **c**, quantitative results of PCBP2 in PC-3 cells under different concentrations of Curcusone C (n = 3); **d**, competitive CC-ABPP silver staining chart; **e**, western blot results of DARTS of binding Curcusone C to PCBP2 protein; **f**, quantitative results of DARTS of binding Curcusone C to PCBP2 protein (n = 3). Compared with the 0 μm control group, *p* > 0.05, **p < 0.01, ***p < 0.001

We next identified proteins that directly interacted with Curcusone C in PC-3 cells using activity-based protein profiling. The probe (final concentration 20 µM) was incubated with PC-3 cells, followed by enrichment and elution. The result of silver staining showed that there was significant difference of the protein bands in the 25–35 kDa between the groups (Fig. 2a). Then, the bands with obvious differences were cut in the 25–35 kDa range for mass spectrometry analysis and identification. We screened out proteins rich in only positive probe group and with peptide coverage > 20%, and arranged them in descending order and obtained the information of top 10 proteins (Table 2).

**Table 2:** Top 10 protein information between 25 and 35 kDa.

Rank	Protein name	Gene name
**1**	Poly(rC)-binding protein 2	PCBP2
**2**	Translational activator of cytochrome c oxidase 1	TACO1
**3**	Adenylate kinase 2, mitochondrial	AK2
**4**	Thioredoxin-dependent peroxide reductase, mitochondrial	PRDX3
**5**	Protein SET	SET
**6**	Peroxiredoxin-4	PRDX4
**7**	Signal recognition particle receptor subunit beta	SRPRB
**8**	Nucleoside diphosphate kinase	NME1-NME2
**9**	Proliferating cell nuclear antigen	PCNA
**10**	Inorganic pyrophosphatase	PPA1

Taking the criteria of high relative abundance and strong association with cancer as the standard, Poly(rC)-binding protein 2 (PCBP2) was the target protein, and other protein information were shown at Supp. Table 1. Then, we used western blot to detect the presence of this protein in PC-3 cells. The results showed that PC-3 cells were rich in PCBP2 protein, and the relative content of PCBP2 decreased with the increase of drug concentration (Fig. 2b, c). In summary, PCBP2 was selected as the active target of Curcusone C.

### Verification of the target protein PCBP2 of Curcusone C

To verify PCBP2 as a target protein of Curcusone C, we first co-incubated Curcusone C and positive probe with PC-3 cells, enabling Curcusone C to competitively bind the sites of positive probe to target protein. The results (Fig. 2d) showed that the protein content in the range of 25–35 kDa in the competition group was lower than the positive probe group.

To further verify PCBP2 as the target of Curcosone C, DARTS experiments were performed. The results of the degree of PCBP2 protein enzymolysis after Curcusone C and PCBP2 protein binding showed when Curcusone C concentration was 100 µM, the amount of enzymolysis by pronase of PCBP2 was lower than that of control group, and the Curcusone C of 100 µM followed, indicating that Curcusone C might reduce the sensitivity of PCBP2 by affecting the spatial conformation of PCBP2 protein after binding with PCBP2 (Fig. 2e, f). The results further proved that Curcusone C had a certain binding effect with PCBP2.

We next verified the direct binding between Curcusone C and PCBP2 using the physical method of surface plasmon resonance. PCBP2 was immobilized onto the CM5 chip and Curcusone C was passed over the chip surface in 5 different concentrations. The interaction between Curcusone C and PCBP2 protein was analyzed based on changes in refractive index. The results (Table 3) showed that the affinity constant between Curcusone C and PCBP2 protein was 3.15 mM, indicating that a certain binding force between Curcusone C and PCBP2 protein.

These results proved that PCBP2 was the target protein of Curcusone C.

**Table 3:** The affinity of curcusone C to PCBP2 using SPR

Method	Ligand	Immobilized level (RU)	Analyte	Analyte Conc.	KD (M)	Rmax (RU)	Chi^2^ (RU^2^)
CM5	PCBP2	17850.3	Curcusone C	31.25–500 µM	3.15E-03	947.7	0.15

### Mechanism of Curcusone C against prostate cancer

To further clarify the mechanism of Curcusone C, we carried out the study on mechanism validation.

**Fig. 3:**
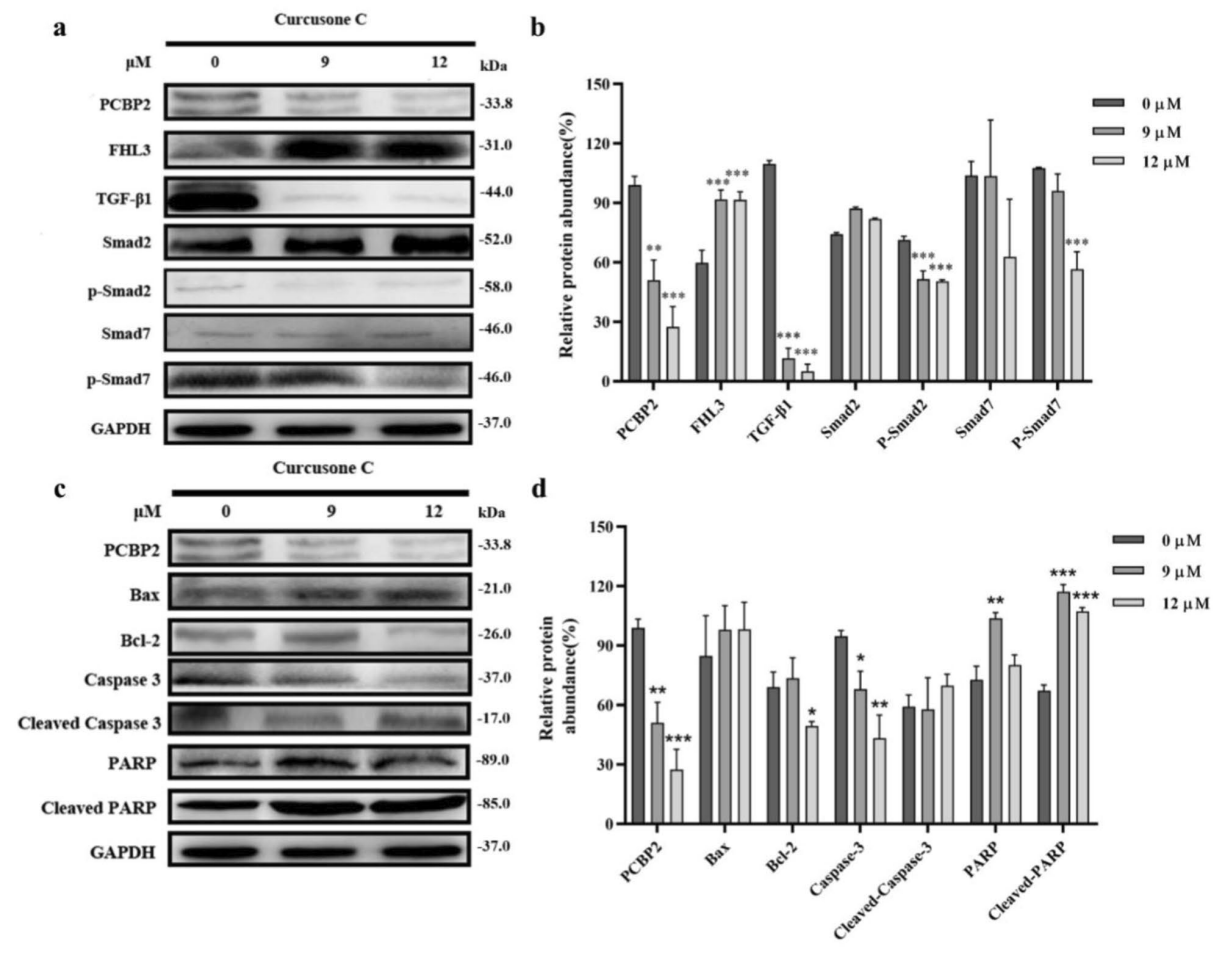
Antitumor pathway of Curcusone C against prostate cancer. **a**, western blot protein bands of Curcusone C on FHL3/TGF-β/Smad signaling pathway; **b**, protein relative expression level of Curcusone C affected FHL3/TGF-β/Smad signaling pathway; **c**, western blot protein bands of Curcusone C on mitochondria apoptosis signaling pathway; **d**, protein relative expression level of Curcusone C affected mitochondria apoptosis signaling pathway. Compared with the 0 µM control group, *p* > 0.05, *p < 0.05, **p < 0.01, ***p < 0.001

Mitochondria also played an important role in apoptosis. In this study, we next analyzed the changes in expression levels of Bax, Bcl-2, caspase-3 and PARP proteins in PC-3 cells treated with Curcusone C. Results as shown in Fig. 3c and d, the relative expression levels of Bax, cleaved caspase-3 and cleaved PARP in PC-3 cells increased gradually with the increase of Curcusone C concentration, while the level of Bcl-2 decreased gradually. Compared with 0 µM Curcusone C group, the activation of PARP in 9 µM and 12 µM Curcusone C group was obviously increased, and the relative expression of Bcl-2 in 12 µM Curcusone C group was remarkably decreased.

These results indicated that CurcusoneC might exert anti-prostate cancer activity by affecting the two above ways: FHL3/TGF-β/Smad signaling pathway and mitochondrial apoptosis pathway.

### Evaluation of curcusone C against prostate cancer in vivo

We further constructed a nude mouse model of prostate cancer by subcutaneous injection of PC-3 cells, evaluated the antitumor activities of Curcusoine C in vivo, and preliminarily explained the in vivo mechanism of Curcusone C against prostate cancer.

**Fig. 4:**
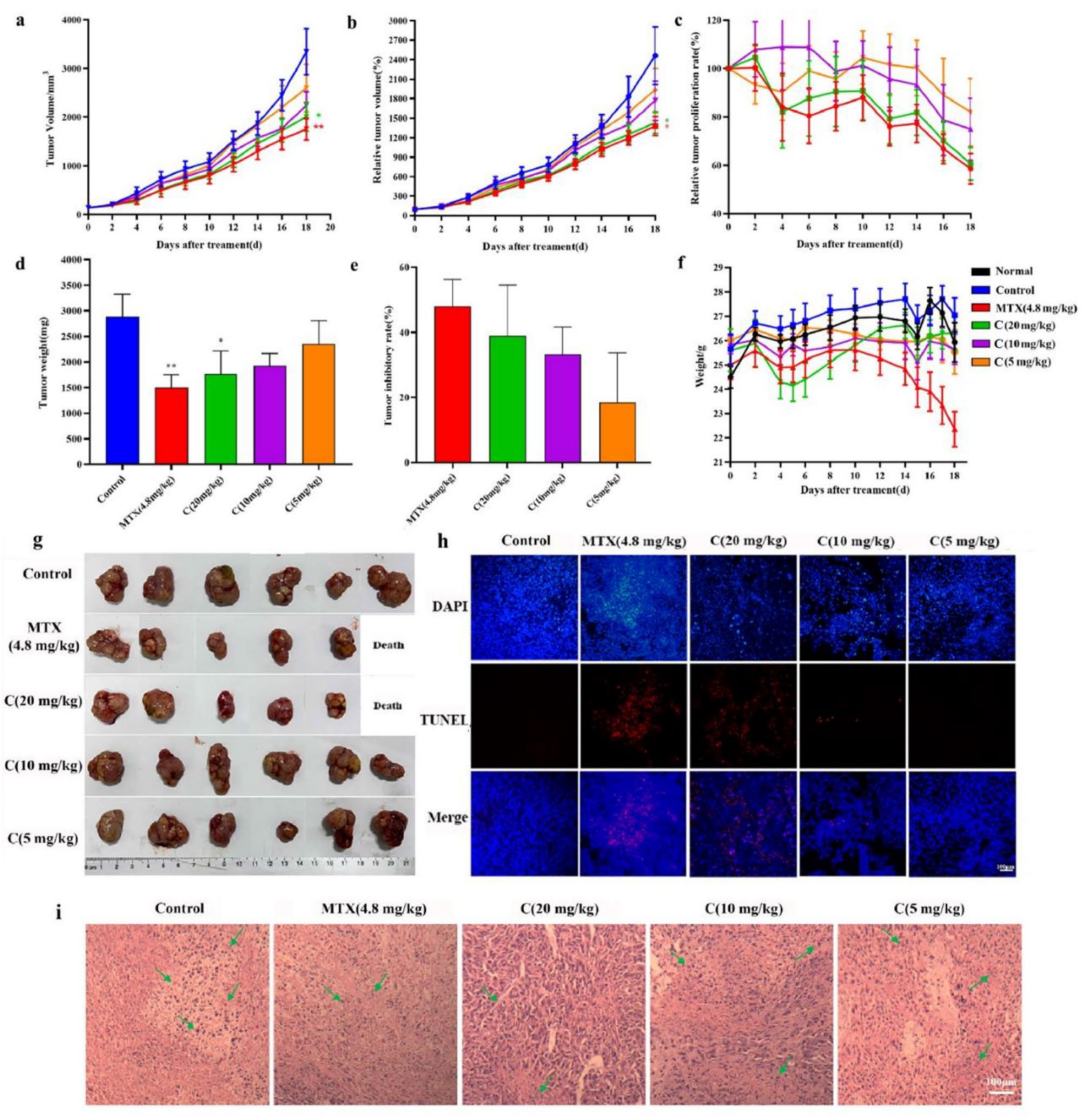
Effects of Curcusone C on tumor in vivo. **a**, effect of drugs on tumor volume in nude mice; **b**, changes of relative tumor volume in nude mice (RTV, %); **c**, changes of relative tumor proliferation rate (%) in nude mice; **d**, effects of drugs on tumor weight in nude mice; **e**, the tumor inhibition rate (%) of drugs; **f**, changes of body weight in nude mice(“C” group represent Curcusone C dosage group); **g**, tumor tissues in the subcutaneous area of nude mice after drug administration; **h**, TUNEL diagram of tumor apoptosis in nude mice (the scale bar 100 μm). The blue signal in DAPI diagram is normal nucleus, and the red signal in TUNEL diagram is apoptotic nucleus; **i**, HE staining of nude mice tumor tissue after drugs administration (the scale bar 100 μm). Green arrow marks tumor tissue apoptosis (nuclear pyknosis, lysis); compared with the control group, *p* > 0.05, *p < 0.05, **p < 0.001, n = 6

In Fig. 4a and b, there were respectively showed the effects of drug injection on tumor volume in nude mice and the changes in relative tumor volume in nude mice. The tumor volume and relative volume of nude mice increased gradually in each group. Compared with the control group, the mean tumor volume of nude mice treated with MTX (4.8 mg/kg) and Curcusone C tended to increase more slowly. Among them, the C (20 mg/kg) group and MTX (4.8 mg/kg) group had the gentlest growth trend, followed by C (10 mg/kg) group and C (5 mg/kg) group. In addition, compared with the control group, the relative tumor proliferation rate of nude mice in the other drug administration groups showed a decreasing trend, among which MTX (4.8 mg/kg) group and C (20 mg/kg) group showed a more obvious decreasing trend (Fig. 4c). On the last day of administration, tumor weight and tumor inhibition rate of nude mice in each group were measured (Fig. 4d). The tumor weight (mg) of nude mice in each group was: control group 2885.5 ± 432.0 mg, MTX group 1503.4 ± 243.0 mg, C (20 mg/kg) group 1759.6 ± 448.7 mg, C (10 mg/kg) group 1925.6 ± 242.1 mg, and C (5 mg/kg) group 2356.7 ± 445.7 mg, in which the average tumor weight difference between C (20 mg/kg) group and control group was 1125.8 mg, indicating that Curcusone C of 20 mg/kg could inhibit the growth of tumor tissue in vivo. Tumor inhibition rates (%) of nude mice in each group were as follows (Fig. 4e): MTX (4.8 mg/kg) group 48%, C (20 mg/kg) group about 39%, C (10 mg/kg) group 33%, C (5 mg/kg) group 18%, indicating MTX and Curcusone C (20 mg/kg) could inhibit the growth of prostate cancer in nude mice to a certain extent. Tumor tissues in the subcutaneous area of nude mice after drug administration were shown in Fig. 4g.

Then, we further performed TUNEL detection (Fig. 4h) and HE staining (Fig. 4i) on the collected tumor tissues. The TUNEL method was used to stain the intracellular deoxyribosomes. It was found that the MTX (4.8 mg/kg) group, C (20 mg/kg) group and C (10 mg/kg) group had red marker signal (representing apoptotic cells), while the control group and C (5 mg/kg) group had no apoptotic cell signal. It is noteworthy that MTX (4.8 mg/kg) group and C (20 mg/kg) group showed the most obvious apoptotic signal, indicating that Curcusone C (20 mg/kg) group had the activity of promoting PC-3 cell apoptosis. The pharmacodynamic activity was similar to that of 4.8 mg/kg MTX in human prostate cancer subcutaneous transplanted tumor model. In addition, HE results showed that a large number of cells in MTX (4.8 mg/kg) and C (20 mg/kg) groups showed nuclear pyknosis and lysis, suggesting that drugs could promote the apoptosis of tumor cells.

**Fig. 5:**
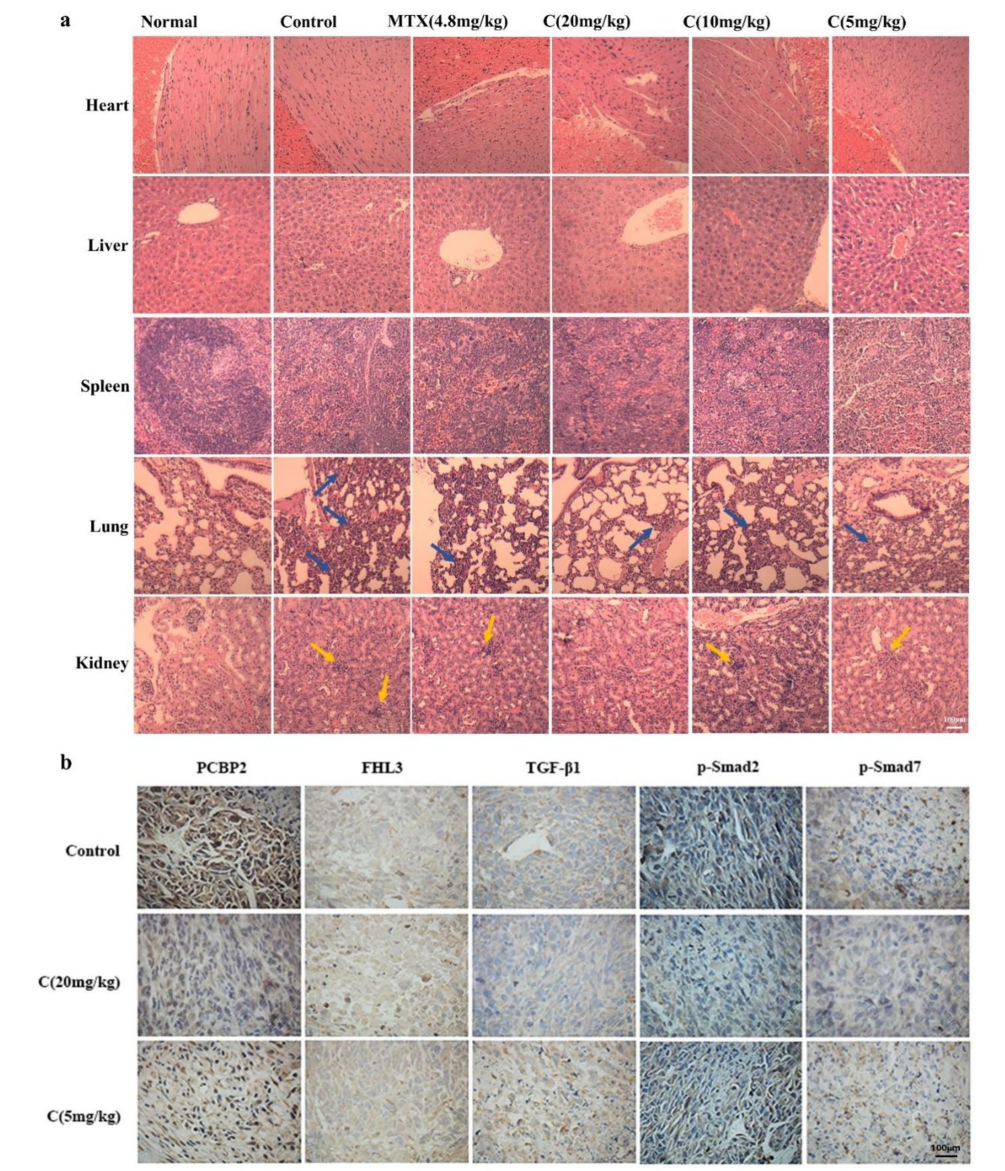
Study on toxicity and antitumor mechanism of Curcusone C in vivo. **a**, HE staining of heart, liver, spleen, lung, and kidney in nude mice (the scale bar 100 μm). The blue arrow marks pulmonary interstitial thickening, and the yellow arrow marks glomerular pyknosis (n = 6); **b**, immunohistochemical results of PCBP2, FHL3, TGF-β1, P-Smad2 and P-Smad7 receptors in nude mouse tumor tissue (the scale bar 100 μm). The brown signals indicate the location and relative expression of the corresponding proteins

The results of body weight of nude mice in each group (Fig. 4f) showed that although there was no statistical difference in body weight among all groups, it could be seen that the fluctuation of body weight of nude mice in all groups tended to be gentle except MTX (4.8 mg/kg) group. Of concern, the weight of nude mice in the Curcusone C group also leveled off during administration. Except for C (20 mg/kg) group, which showed a weight loss due to intolerance at the initial stage of drug administration, the body weight of nude mice gradually adjusted to the drug and then stabilized. Then, to further study the toxicity of Curcusone C, heart, liver, spleen, lung and kidney of nude mice in each group were stained with HE. As shown in Fig. 5a, the five organs of the nude mice treated with Curcusone C showed no significant changes compared with the normal group, and the lung tissues of the control group and MTX (4.8 mg/kg) group showed significant pulmonary interstitial thickening and pneumonic pathological manifestations. Excluding the influence of the feeding environment, we hypothesized that this was caused by the action of cancer cells. C (20 mg/kg) showed no interstitial thickening of lung tissue, indicating that Curcusone C could improve lung injury caused by tumor to a certain extent and had lung protection function. In addition, the glomerulus in the renal cortex of nude mice in the MTX (4.8 mg/kg) group showed severe pyknosis, while there was no obvious abnormality in the renal tissue after administration of Curcusone C.

In addition, combined with the results of in vitro mechanism experiments, we selected PCBP2, FHL3, TGF-β1, phosphorylated Smad2 and phosphorylated Smad7 as the research objects of anti-tumor mechanism in vivo, and immunohistochemistry was used to detect the changes of protein expression in tumor tissues after drug treatment. The results (Fig. 5b) showed that the expression of PCBP2 protein in tumor tissues decreased with the increase of drug concentration; compared with the control group, the protein content in C group (20 mg/kg) was decreased; the content of FHL3 protein in C (20 mg/kg) group was higher than that in Control group; the changes of TGF-β1, P-Smad2 and P-smad7 proteins were similar to those of PCBP2, and all showed a downward trend. These results further validated the mechanism of Curcusone C against prostate cancer.

## Discussion

Curcusones series was a key antitumor active substance in *J. carcass*. In our previous studies, the results of Curcusones series of compounds incubated with cancer cells in three major systems (the digestive, reproductive and circulatory systems) displayed that Curcusone C had a very good inhibitory effect on human prostate PC-3 cells, indicating Curcusone C might have good anti-prostate cancer activity.

Our previous studies showed that the dienone structure fragment of the B ring is the active center of the Curcusones series of compounds [15]. Therefore, in this study, we next synthesized the chemical probes keeping the B-ring structure unchanged and evaluated the activities of probes. Then, we used the probes to explore the key target protein interacted with Curcusone C.

Through collecting the corresponding proteins with significant differences between positive and negative probes, we identified them by mass spectrometry and nearly 4000 known proteins were matched by database comparison. Results displayed that PCBP2 ranked first and was most correlated with cancer. Interestingly, PCBP2 was an RNA-binding protein that was involved in regulating and stabilizing the mRNA translation of cancer-related protein in tumor cells, and further regulating the development of cancer [16]. In addition, Wen and colleagues used cervical cancer cells to find that PCBP2 interacts with long non-coding RNA to stabilize RRM1 mRNA and promote epithelial mesenchymal transition and proliferation of cancer cells [17]. Chen et al. found that PCBP2 was a promoter of gastric cancer in gastric cells. The protein content in cancer tissues was remarkably higher than that in normal tissues. However, when PCBP2 was silenced, the proliferation capacity of HGC-27 cells was obviously reduced, suggesting that PCBP2 could be a potential target for gastric cancer treatment [18]. These studies suggested that PCBP2 was the most likely target of Curcusone C for its anti-prostate cancer activity.

In order to verify the hypothesis that PCBP2 protein was the target protein of Curcusone C, Curcusone C and the positive probe were used to compete with each other. Through competitive difference analysis and verification, PCBP2 protein was indeed a differential protein. Then, we found that Curcusone C could reduce the degree of enzymatic hydrolysis of PCBP2 protein through DARTS experiment. Finally, SPR assay results showed that Curcusone C had certain binding ability with PCBP2 protein. These results supported our hypothesis that PCBP2 was an active target protein of Curcusone C.

By the way, it is remarkable that in previous studies we synthesized the probe based on Cursosone D and used it to discover that BRAT1 that is a master regulator of the DDR and DNA repair might be the key target oncoprotein of curcusone D in Hela cells [19]. Interestingly, the only difference between A and B is that the C-2 configuration of compounds is different, indicating that the difference of configuration is an indispensable factor to be considered in target identification. Meanwhile, our previous findings suggest that BRAT1 may also be a potential protein for curcusone C to exert anti-prostate cancer activity, but this idea needs further proof.

In addition to target identification and verification, we also further studied the mechanism of action. Previous studies had shown that Curcusones analogs mediated cancer cell death through multiple pathways [10, 11, 20]. To further understand how Curcusone C exerted activities, we validated the mechanism of anti-prostate cancer of Curcusone C. Four and a half LIM domain protein 3 (FHL3) was associated with inhibition of cell apoptosis. Studies had shown that when PCBP2 was knocked out, the expression of FHL3 in cancer cells was increased, thus inhibiting cell growth and promoting cell apoptosis [16]. Our results indicated that Curcusone C can inhibit the proliferation and metastasis in prostate cancer cells through activating the expression of FHL3 and repressing PCBP2 through suppressing TGF/Smad signaling pathway, and the result was similar to studies of Mao and colleagues [21]. In addition, mitochondria could induce endogenous apoptosis by releasing signal factors to activate caspase. For example, the imbalance of the ratio of Bcl-family protein Bax/Bcl-2 would cause changes in mitochondrial membrane structure and induce the release of cytochrome C, thus activating the caspase cascade and promoting the apoptosis process of cells [22–24]. Related studies had shown that when PCBP2 was knocked out, the expression of pro-apoptotic factor Bax was increasing and anti-apoptotic factor Bcl-2 was decreasing, and caspase-3 and PARP were activated to mediate apoptosis of cancer cells [16, 25]. Our results suggested that Curcusone C disrupt the Bax/Bcl-2 balance in PC-3 cells by inhibiting the expression of the target protein PCBP2, thereby inducing mitochondrial damage and activation of the mitochondrial apoptosis pathway, and ultimately inducing apoptosis of prostate cancer cells, which was similar with the research of Zhang et al. as mentioned before [11]. However, further researches are needed to determine the specific causal relationship and direct effect of PCBP2-mediated FHL3/TGF-β1/Smad signaling pathway and mitochondrial apoptosis pathway through cell model or related inhibitors and immunoprecipitation techniques, and determine the complete mechanism pathway and the domain of interaction between small molecules and proteins.

We finally constructed a nude mouse model of prostate cancer, and evaluated the antitumor activity of Curcusone C and had further insight into the mechanism in vivo. The results suggested that 20 mg/kg Curcusone C had the pharmacodynamic activity to inhibit the proliferation of subcutaneous transplanted tumor models of prostate cancer and was similar to the activity of 4.8 mg/kg methotrexate. In addition, the body weight of nude mice in the C (20 mg/kg) group showed a stable trend and no significant changes in organs, indicating that the toxicity of Curcusone C to normal cells was less than that of methotrexate. At last, immunohistochemical results further proved that Curcusone C induced the decreasing expression of PCBP2, TGF-β1, P-Smad2 and P-smad7 and increasing expression of FHL3 in nude mouse tumors, and inhibited tumor cell growth, proliferation and metastasis in nude mice.

## Conclusions

In conclusion, this study demonstrated that Curcusone C can target PCBP2 protein, thereby affecting TGF/Smad signaling pathway and Bax/Bcl-2 balance, and ultimately exert anti-prostate cancer activity. our study provides an effective basis for the development of triterpenes, as well as the discovery and use of novel anti-prostate cancer drugs with stronger targeting, better activity, less toxic and side effects and stronger selectivity.

### Electronic supplementary material

Below is the link to the electronic supplementary material.

Supplementary Material 1

## Data Availability

All data are available from the corresponding author upon reasonable request.
