# Supplementary material for: Unveiling poly(rC)-binding protein 2 as the target protein for curcusone C against prostate cancer: mechanism validation through click chemistry-activity based proteomics profiling approach
Source: BMC Cancer. 2023 Oct 9;23:957. doi: 10.1186/s12885-023-11467-0 (PMC10563230; doi:10.1186/s12885-023-11467-0)
Supplement: Supplementary file 1 — Supplementary Material 1 [file 12885_2023_11467_MOESM1_ESM.docx]

**
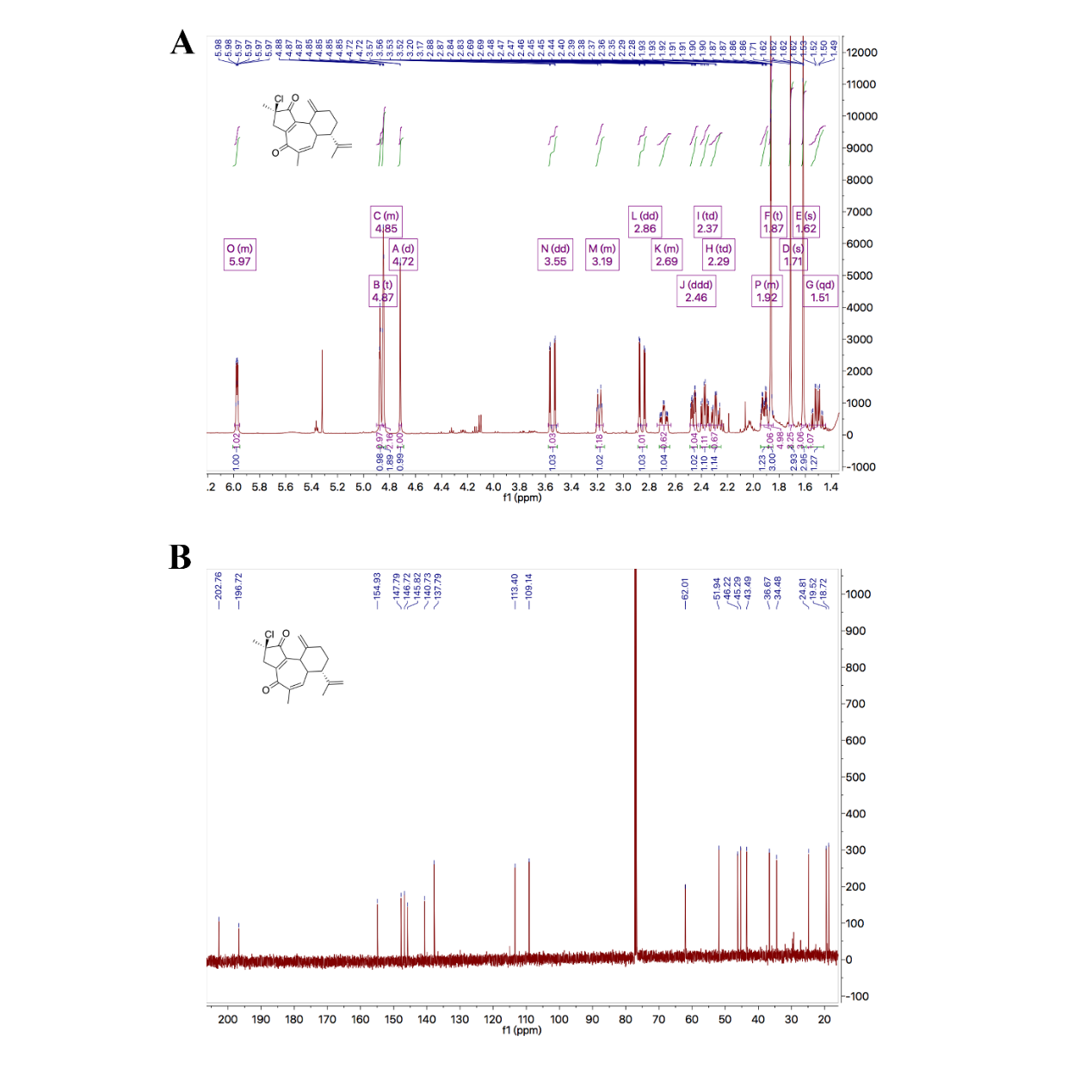
Supplementary Figure 1. Structural information of derivative C-1.** ESI-MS m/z 353.13 [M + Na]^+^. A, ^1^H-NMR (CDCl_3_, 500 MHz) *δ* : 2.86 (1H, dd, *J* = 19.3, 3.5 Hz, H-3α), 3.55 (1H, dd, *J* = 19.3, 2.7 Hz, H-3β), 5.97 (1H, m, H-7), 2.69 (1H, m, H-8), 3.19 (1H, m, H-9), 2.37 (1H, td, *J* = 11.9, 4.2 Hz, H-12α), 2.29 (1H, td, *J* = 12.7, 5.0 Hz, H-12β), 1.92 (1H, m, H-13α), 1.51 (1H, qd, *J* = 12.8, 4.5 Hz, H-13β), 2.46 (1H, ddd, *J* = 12.7, 4.5, 2.7 Hz, H-14), 4.87 (1H, d, *J* = 1.5 Hz, H-16α), 4.72 (1H, d, *J* = 1.2 Hz, H-16β), 1.71 (1H, s, H-17), 4.85 (2H, s, H-18), 1.62 (3H, s, H-19), 1.87 (3H, t, 1.7, H-20); B, ^13^C-DEPT NMR (CDCl_3_, 125 MHz) *δ*C: 202.76 (C-1), 62.01 (C-2), 43.49 (C-3), 154.93 (C-4), 196.72 (C-5), 140.73 (C-6), 137.79 (C-7), 45.29 (C-8), 46.22 (C-9), 145.82 (C-10), 147.79 (C-11), 34.48 (C-12), 36.67 (C-13), 51.94 (C-14), 146.72 (C-15), 113.40 (C-16), 18.72 (C-17), 109.14 (C-18), 24.81 (C-19), 19.52 (C-20).


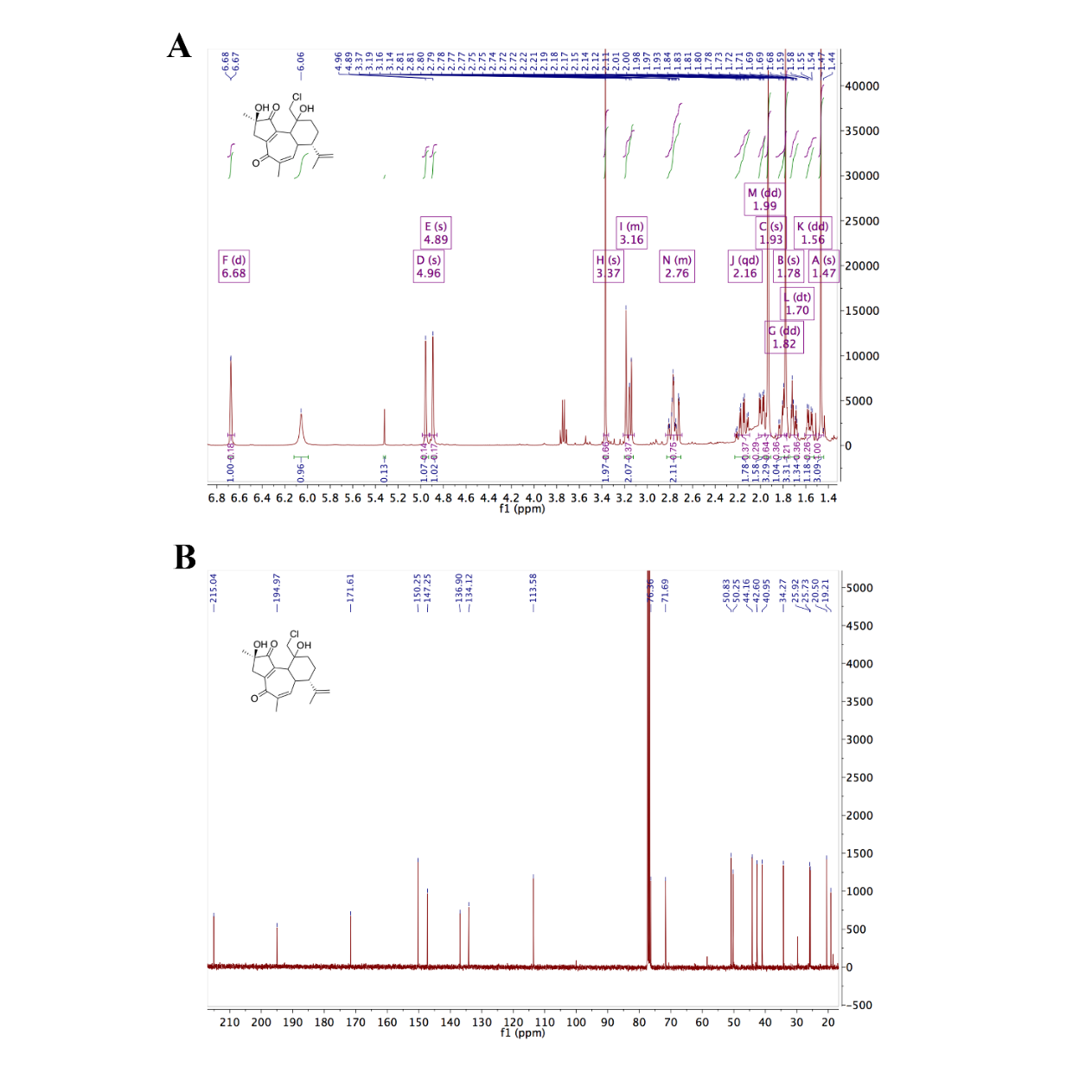
**Supplementary Figure 2. Structural information of derivative C-2.** ESI-MS m/z 387.13 [M + Na]^+^. A, ^1^H-NMR (CDCl_3_, 500 MHz) *δ* : 2.76 (1H, m, H-3α), 3.16 (1H, m, H-3β), 6.68 (1H, m, H-7), 2.76 (1H, m, H-8), 3.16 (1H, m, H-9), 2.16 (2H, qd, *J* = 13.0, 3,8 Hz, H-12), 1.56 (1H, dd, *J* = 13.0, 3.7 Hz, H-13α), 1.82 (1H, dd, *J* = 13.4, 3.6 Hz, H-13β), 1.70 (1H, dt, *J* = 13.4, 3.5 Hz, H-14), 4.96 (1H, s, H-16α), 4.89 (1H, s, H-16β), 1.78 (3H, s, H-17), 3.37 (2H, s, H-18), 1.47 (3H, s, H-19), 1.93 (3H, s, H-20); B, ^13^C-DEPT NMR (CDCl_3_, 100 MHz) *δ*C: 215.04 (C-1), 76.36 (C-2), 42.60 (C-3), 171.61 (C-4), 194.97 (C-5), 134.12 (C-6), 150.25 (C-7), 40.95 (C-8), 44.16 (C-9), 136.90 (C-10), 71.69 (C-11), 34.27 (C-12), 25.73 (C-13), 50.83 (C-14), 147.25 (C-15), 113.58 (C-16), 19.21 (C-17), 50.25 (C-18), 25.73 (C-19), 20.50 (C-20).


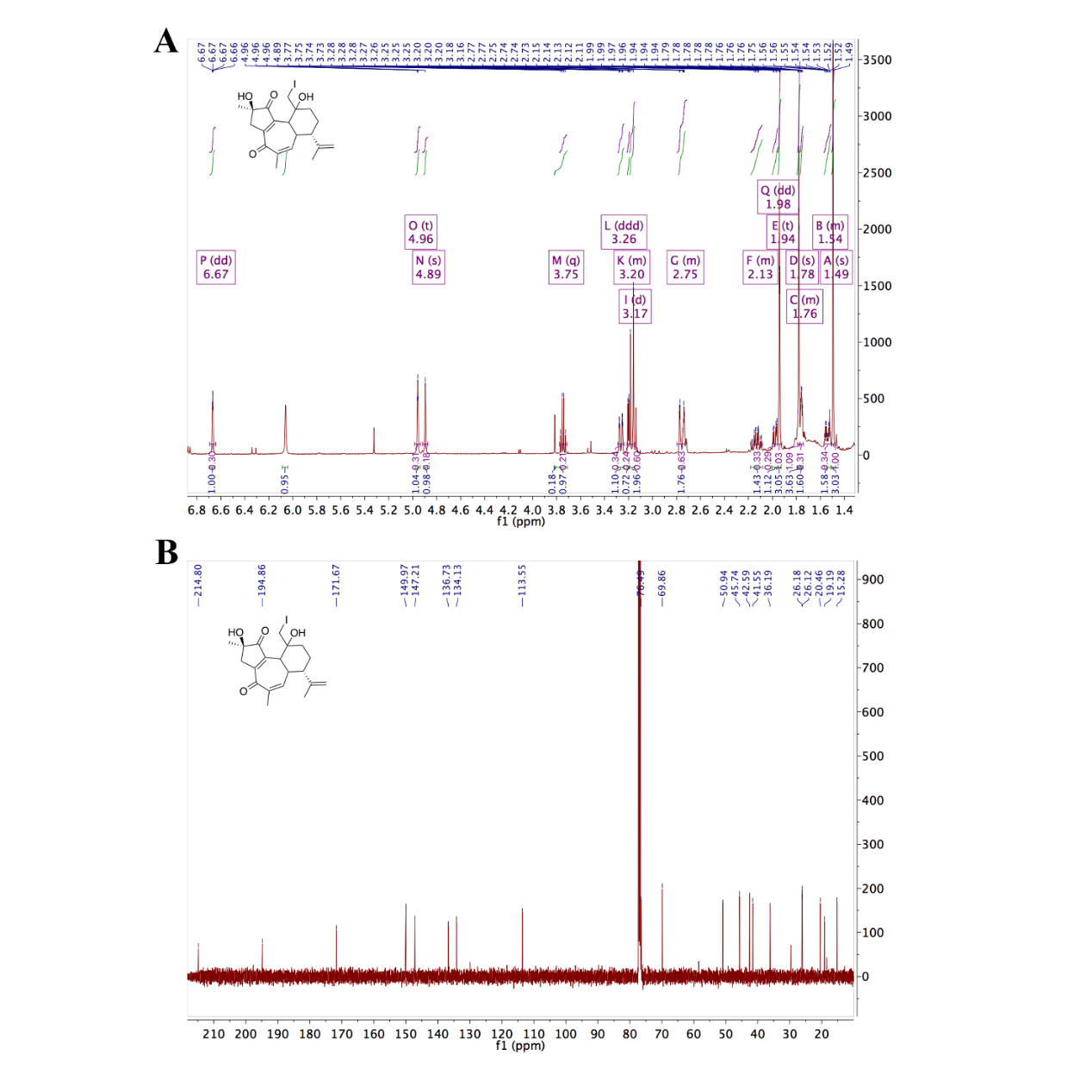
**Supplementary Figure 3. Structural information of derivative C-3.** ESI-MS m/z 479.07 [M + Na]^+^. A, ^1^H-NMR (CDCl_3_, 500 MHz) δ : 2.75 (2H, m, H-3), 6.67 (1H, m, H-7), 3.26 (1H, ddd, *J* = 12.2, 2.3, 1.2 Hz, H-8), 3.20 (1H, m, H-9), 1.76 (2H, m, H-12), 2.13 (1H, m, H-13α), 1.54 (1H, m, H-13β), 1.98 (1H, dd, *J* = 11.8, 4.1 Hz, H-14), 4.96 (1H, s, H-16a), 4.89 (1H, s, H-16b), 1.78 (3H, s, H-17), 3.17 (2H, d, *J* = 12.1 Hz, H-18), 1.49 (3H, s, H-19), 1.94 (3H, t, *J* = 1.6 Hz, H-20); B, ^13^C-DEPT NMR (CDCl_3_, 125 MHz) δC: 214.80 (C-1), 76.49 (C-2), 42.59 (C-3), 171.67 (C-4), 194.86 (C-5), 134.13 (C-6), 148.97 (C-7), 41.55 (C-8), 45.74 (C-9), 136.73 (C-10), 69.86 (C-11), 36.19 (C-12), 26.18 (C-13), 50.94 (C-14), 147.21 (C-15), 113.55 (C-16), 19.19 (C-17), 15.28 (C-18), 26.12 (C-19), 20.46 (C-20).


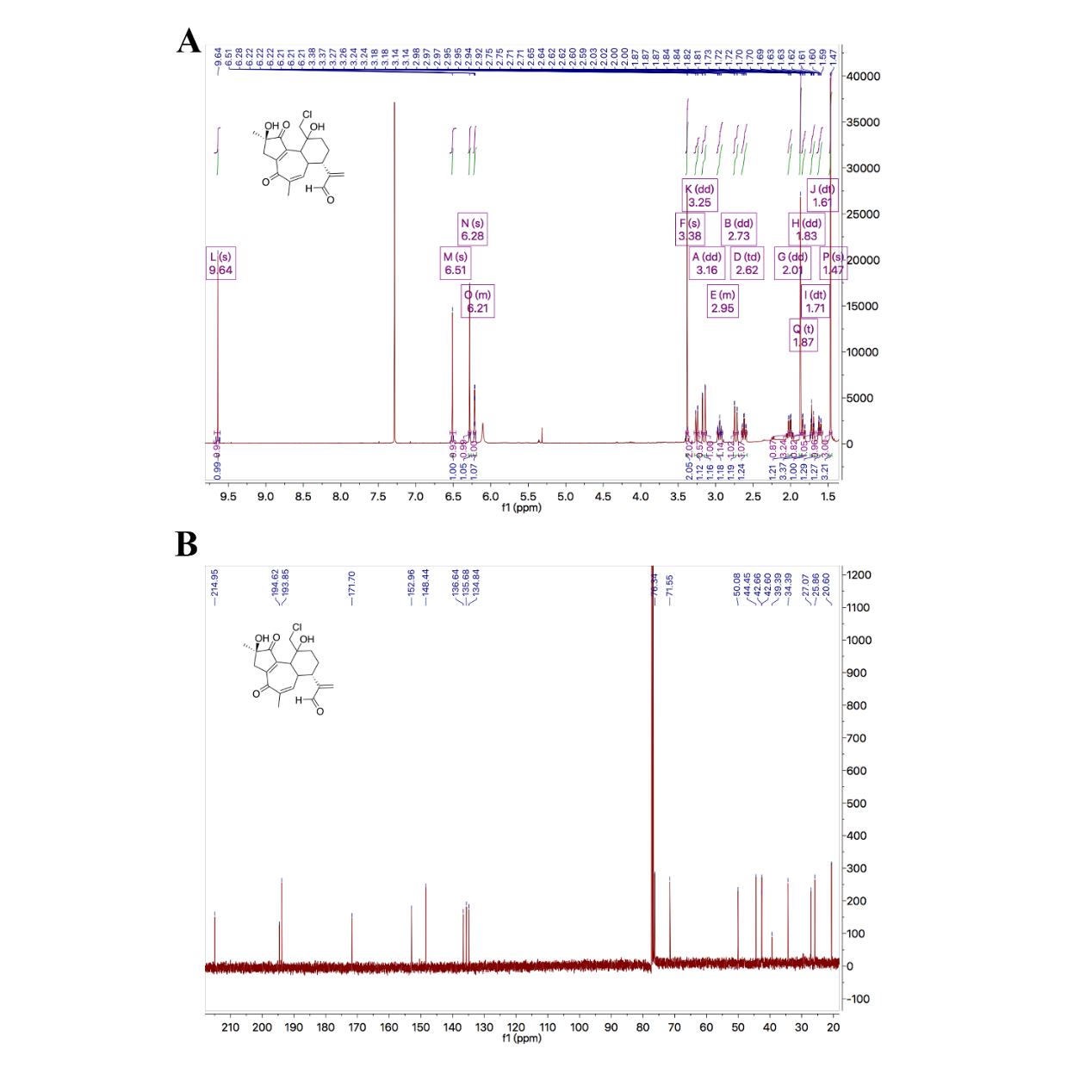
**Supplementary Figure 4. Structural information of derivative C-4.** ESI-MS m/z 401.11 [M + Na]^+^. A, ^1^H-NMR (CDCl_3_, 500 MHz) *δ* : 2.73 (1H, dd, *J* = 18.9, 2.2 Hz, H-3*α*), 3.16 (1H, dd, *J* = 18.9, 1.2 Hz, H-3*β*), 6.21 (1H, m, H-7), 2.62(1H, td, *J* = 12.2, 4.2 Hz, H-8), 2.95 (1H, m, H-9), 2.01 (1H, dd, *J* = 12.9, 3.5 Hz, H-12*α*), 1.83 (1H, dd, *J* = 13.4, 3.6 Hz, H-12*β*), 1.71 (1H, dt, *J* = 13.5, 3.4 Hz, H-13*α*), 1.61 (1H, dt, *J* = 13.0, 3.7 Hz, H-13*β*), 3.25 (1H, dd, *J* = 12.2, 1.1 Hz, H-14), 6.51 (1H, s, H-16*α*), 6.28 (1H, s, H-16*β*), 9.64 (1H, s, H-17), 3.38 (2H, s, H-18), 1.47 (3H, s, H-19), 1.95 (3H, t, *J* = 1.7 Hz, H-20); B, ^13^C-DEPT NMR (CDCl_3_, 125 MHz) *δ*_C_: 214.95 (C-1), 76.34 (C-2), 42.66 (C-3), 171.70 (C-4), 194.62 (C-5), 135.68 (C-6), 152.96 (C-7), 39.39 (C-8), 44.45 (C-9), 136.64 (C-10), 71.55 (C-11), 34.39 (C-12), 27.07 (C-13), 42.60 (C-14), 148.44 (C-15), 134.84 (C-16), 193.85 (C-17), 50.08 (C-18), 25.86 (C-19), 20.60 (C-20).


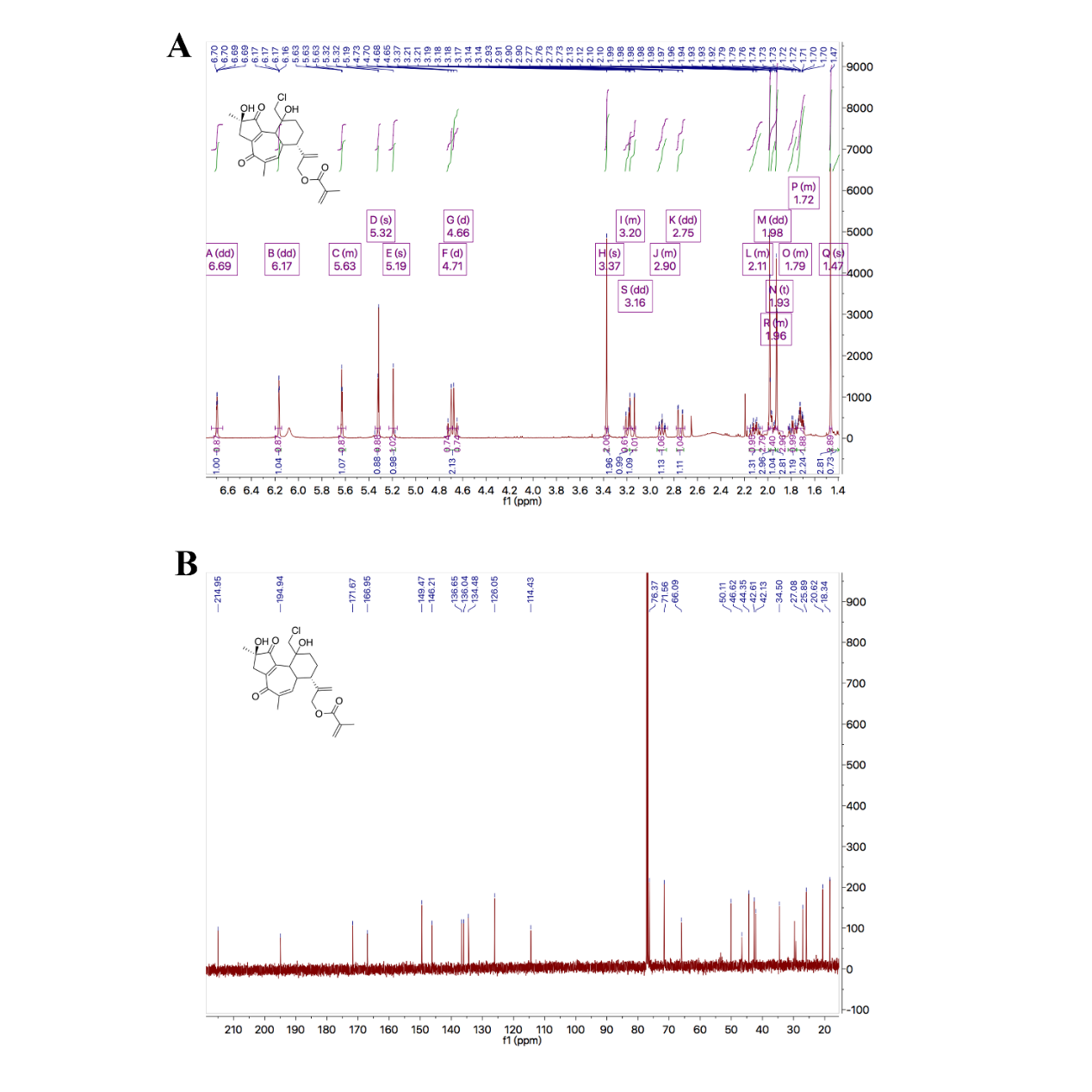
**Supplementary Figure 5. Structural information of derivative C-5.** ESI-MS m/z 445.14 [M + Na]^+^. A, ^1^H-NMR (CDCl_3_, 500 MHz) *δ* : 2.75 (1H, dd, *J* = 18.9, 2.1 Hz, H-3*α*), 3.16 (1H, dd, *J* = 18.8 Hz, 1.2, H-3*β*), 5.63 (1H, m, H-7), 2.90 (1H, m, H-8), 3.20 (1H, m, H-9), 1.97 (1H, m, H-12*α*), 1.79 (1H, m, H-12*β*), 1.72 (2H, m, H-13), 2.11 (1H, m, H-14), 5.32 (1H, s, H-16*α*), 5.19 (1H, s, H-16*β*), 4.71 (1H, d, *J* = 13.5 Hz, H-17*α*), 4.63 (1H, d, *J* = 13.6 Hz, H-17*β*), 3.37 (2H, s, H-18), 1.47 (3H, s, H-19), 1.93 (3H, t, *J* = 1.6 Hz, H-20), 6.69 (1H, dd, *J* = 2.5, 1.3 Hz, H-22), 6.17 (1H, dd, *J* = 1.6, 0.9 Hz, H-22), 1.98 (3H, dd, *J* = 1.6, 1.0 Hz, H-24); B, ^13^C-DEPT NMR (CDCl_3_, 125 MHz) *δ*_C_: 214.95 (C-1), 76.37 (C-2), 42.61 (C-3), 171.67 (C-4), 194.94 (C-5), 134.48 (C-6), 149.47 (C-7), 42.31 (C-8), 44.35 (C-9), 136.65 (C-10), 71.56 (C-11), 34.50 (C-12), 27.28 (C-13), 46.62 (C-14), 146.21 (C-15), 114.43 (C-16), 66.09 (C-17), 50.11 (C-18), 25.89 (C-19), 20.62 (C-20), 166.95 (C-21), 136.04 (C-22), 126.05 (C-23), 18.34 (C-24).


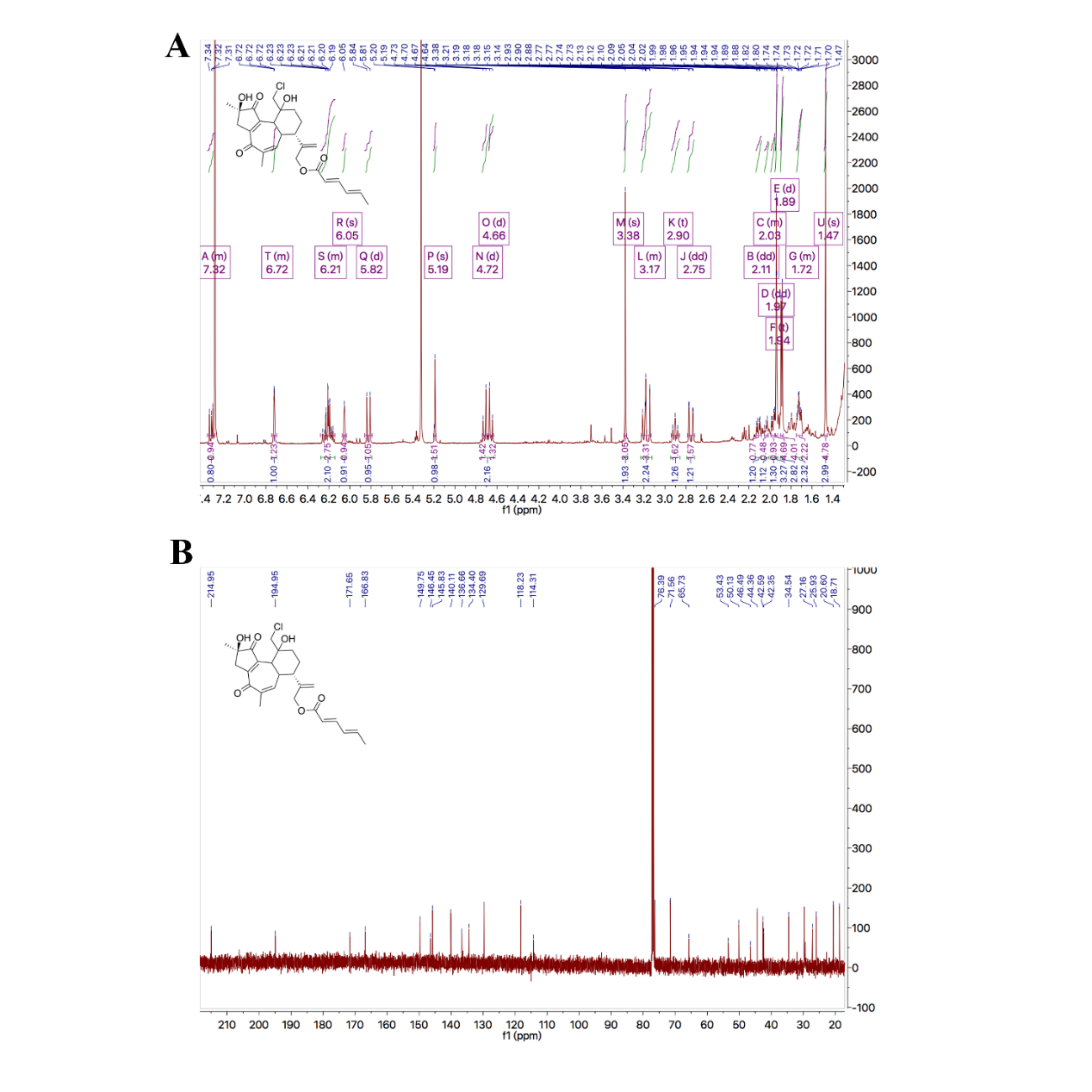
**Supplementary Figure 6. Structural information of derivative C-6.** ESI-MS m/z 497.17 [M + Na]^+^. A, ^1^H-NMR (CDCl_3_, 500 MHz) *δ* : 2.75 (1H, dd, *J* = 18.8, 2.1 Hz, H-3*α*), 3.17 (1H, m, H-3*β*), 6.72 (1H, m, H-7), 2.90 (1H, t, *J* = 12.0 Hz, H-8), 3.17 (1H, m, H-9), 2.11 (1H, dd, *J* = 12.8, 3.9 Hz, H-12*α*), 1.97 (1H, dd, *J* = 12.1, 3.7 Hz, H-12*β*), 1.72 (2H, m, H-13), 2.03 (1H, m, H-14), 6.05 (1H, s, H-16*α*), 5.19 (1H, s, H-16*β*), 4.72 (1H, d, *J* = 13.6 Hz, H-17*α*), 4.66 (1H, d, *J* = 13.6 Hz, H-17*β*), 3.38 (2H, s, H-18), 1.47 (3H, s, H-19), 1.94 (3H, t, *J* = 1.6 Hz, H-20), 5.82 (1H, d, *J* = 15.6 Hz, H-22), 7.32 (1H, m, H-23), 6.21 (1H, m, H-24), 6.21 (1H, m, H-25), 1.89 (3H, d, *J* = 5.8 Hz, H-26); B, ^13^C-DEPT NMR (CDCl_3_, 125 MHz) *δ*_C_: 214.95 (C-1), 76.39 (C-2), 42.59 (C-3), 171.65 (C-4), 194.95 (C-5), 134.40 (C-6), 149.75 (C-7), 42.35 (C-8), 44.36 (C-9), 136.66 (C-10), 71.56 (C-11), 34.54 (C-12), 27.16 (C-13), 46.49 (C-14), 146.45 (C-15), 114.31 (C-16), 65.73 (C-17), 50.13 (C-18), 25.93 (C-19), 20.60 (C-20), 166.83 (C-21), 118.23 (C-22), 145.83 (C-23), 129.69 (C-24), 140.11 (C-25), 18.71 (C-26).


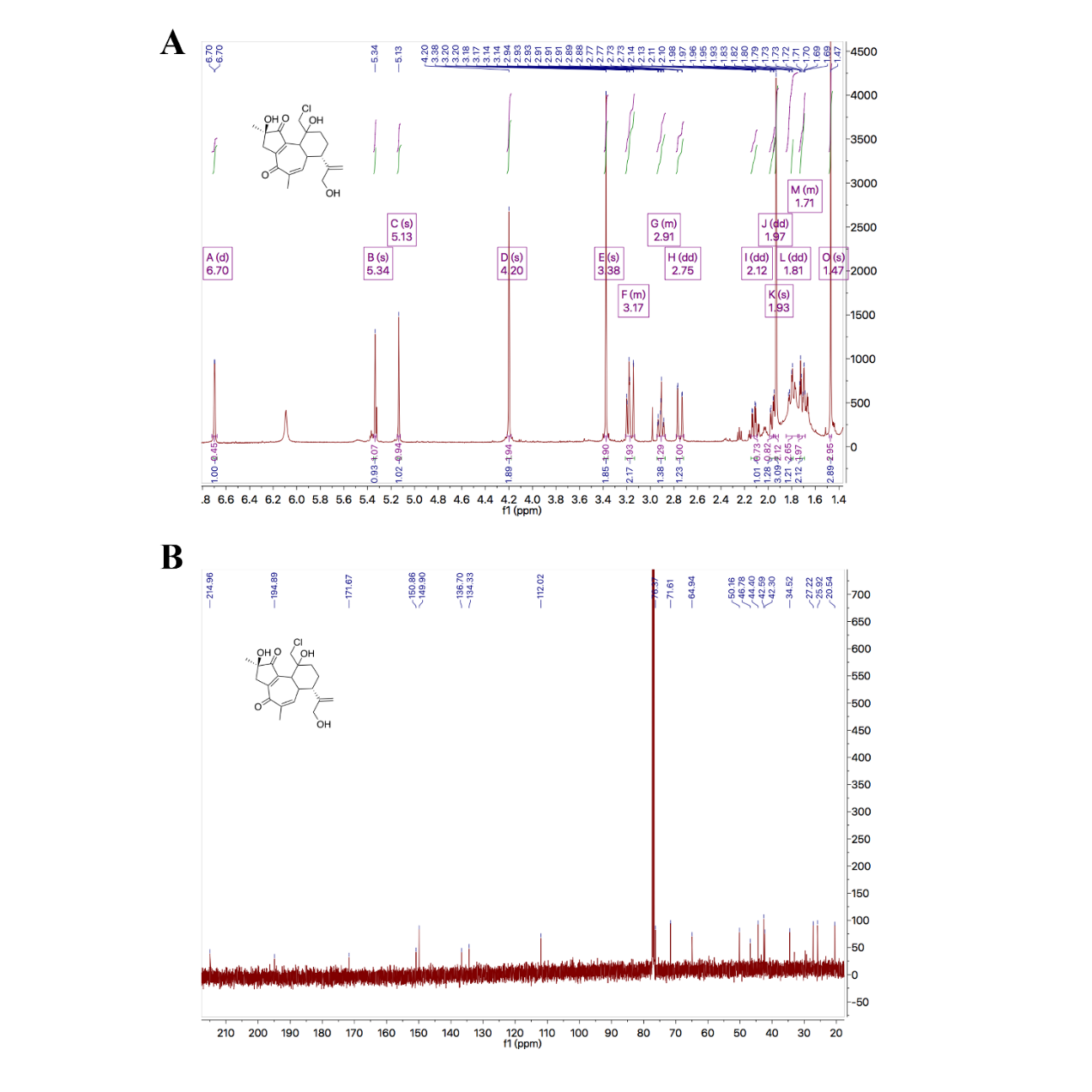
**Supplementary Figure 7. Structural information of derivative C-7.** ESI-MS m/z 403.13 [M + Na]^+^. A, ^1^H-NMR (CDCl_3_, 500 MHz) *δ* : 2.91 (1H, m, H-3*α*), 3.17 (1H, m, H-3*β*), 6.70 (1H, d, *J* = 1.1 Hz, H-7), 3.17 (1H, m, H-8), 2.75 (1H, dd, *J* = 18.9, 2.2 Hz, H-9), 2.12 (1H, dd, *J* = 12.7, 3.5 Hz, H-12*α*), 1.80 (1H, dd, *J* = 13.2, 3.6 Hz, H-12*β*), 1.71 (2H, m, H-13), 1.97 (1H, dd, *J* = 11.9, 4.0 Hz, H-14), 5.34 (1H, s, H-16*α*), 5.13 (1H, s, H-16*β*), 4.20 (2H, s, H-17), 3.38 (2H, s, H-18), 1.47 (3H, s, H-19), 1.93 (3H, s, H-20); B, ^13^C-DEPT NMR (CDCl_3_, 125 MHz) *δ*_C_: 214.96 (C-1), 76.37 (C-2), 42.59 (C-3), 171.67 (C-4), 194.89 (C-5), 134.33 (C-6), 149.90 (C-7), 42.30 (C-8), 44.40 (C-9), 136.70 (C-10), 71.61 (C-11), 34.52 (C-12), 27.22 (C-13), 46.78 (C-14), 150.86 (C-15), 112.02 (C-16), 64.94 (C-17), 50.16 (C-18), 25.92 (C-19), 20.54 (C-20).


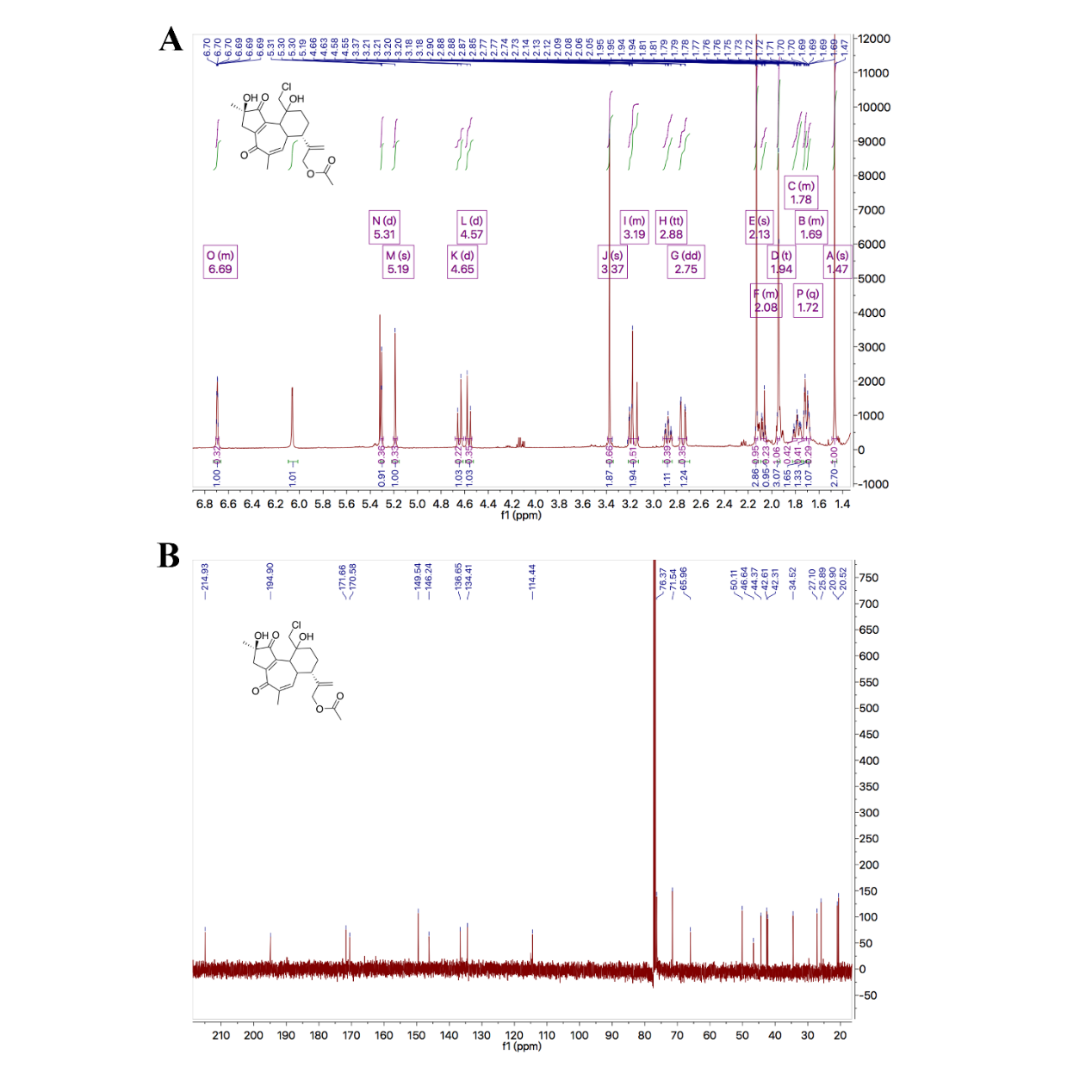
**Supplementary Figure 8. Structural information of derivative C-8.** ESI-MS m/z 445.14 [M + Na]^+^. A, ^1^H-NMR (CDCl_3_, 500 MHz) *δ* : 2.88 (1H, m, H-3*α*), 3.19 (1H, m, H-3*β*), 6.69 (1H, m, H-7), 3.17 (1H, m, H-8), 2.75 (1H, dd, *J* = 18.9, 2.1 Hz, H-9), 1.78 (2H, m, H-12), 1.72 (1H, m, H-13*α*), 1.69 (1H, m, H-13*β*), 2.08 (1H, m, H-14), 5.31 (1H, s, H-16*α*), 5.19 (1H, s, H-16*β*), 4.65 (1H, d, *J* = 13.6 Hz, H-17), 4.57 (1H, d, *J* = 13.6 Hz, H-17), 3.37 (2H, s, H-18), 1.47 (3H, s, H-19), 1.94 (3H, t, *J* = 1.7 Hz, H-20); B, ^13^C-DEPT NMR (CDCl_3_, 125 MHz) *δ*_C_: 214.93 (C-1), 76.37 (C-2), 42.61 (C-3), 171.66 (C-4), 194.90 (C-5), 134.41 (C-6), 149.54 (C-7), 42.31 (C-8), 44.37 (C-9), 136.65 (C-10), 71.54 (C-11), 34.52 (C-12), 27.10 (C-13), 46.64 (C-14), 146.24 (C-15), 114.44 (C-16), 65.96 (C-17), 50.11 (C-18), 25.89 (C-19), 20.52 (C-20), 170.58 (C-21), 20.90 (C-22).


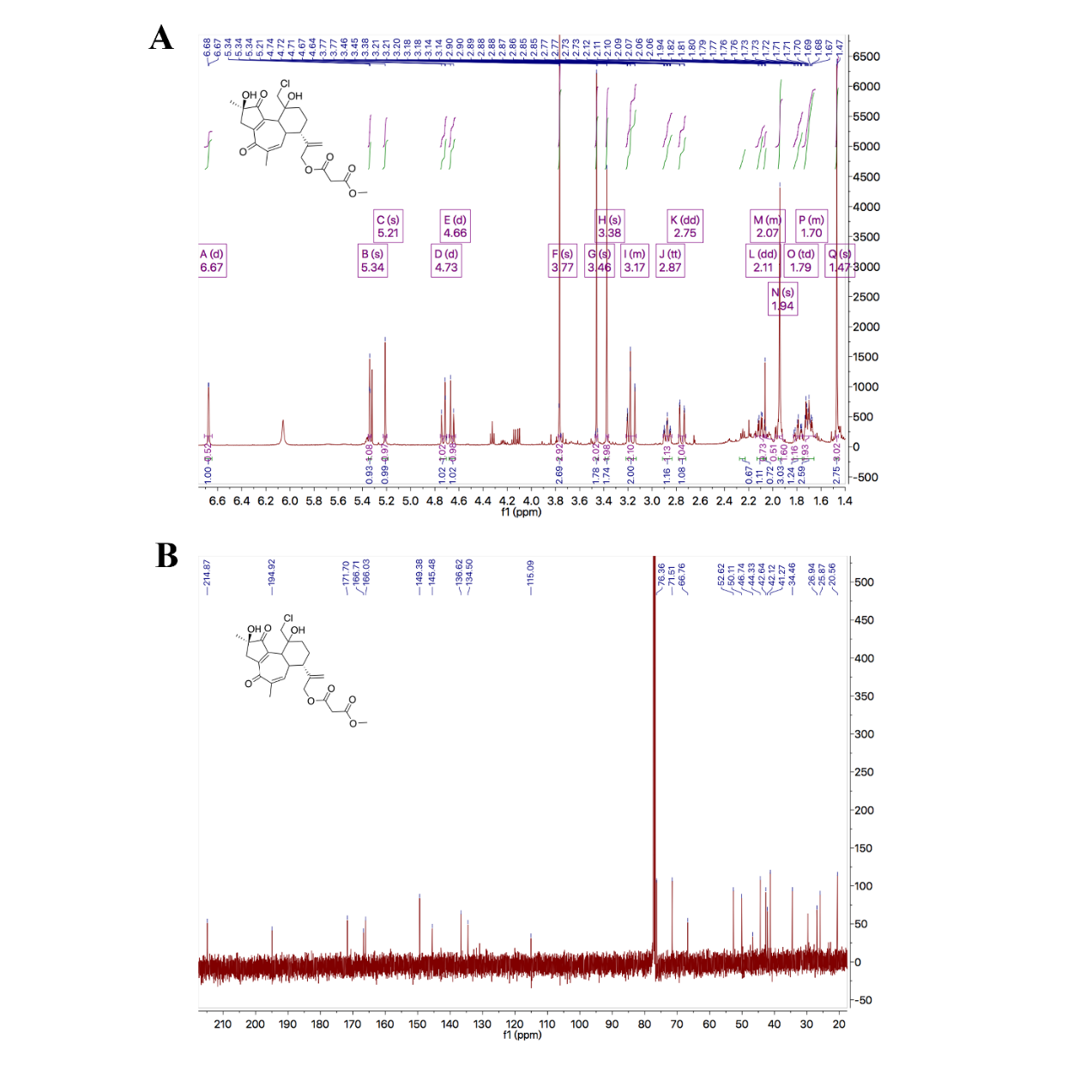
**Supplementary Figure 9. Structural information of derivative C-9.** ESI-MS m/z 503.15 [M + Na]^+^. A, ^1^H-NMR (CDCl_3_, 500 MHz) *δ* : 2.87 (1H, m, H-3*α*), 3.17 (1H, m, H-3*β*), 6.68 (1H, m, H-7), 2.75 (1H, dd, *J* = 19.0, 2.2 Hz, H-8), 3.17 (1H, m, H-9), 2.07 (1H, m, H-12*α*), 1.79 (1H, m, H-12*β*), 1.70 (2H, m, H-13), 2.11 (1H, dd, *J* = 13.0, 3.8 Hz, H-14), 5.34 (1H, s, H-16*α*), 5.21 (1H, s, H-16*β*), 4.73 (1H, d, *J* = 13.5 Hz, H-17*α*), 4.66 (1H, d, *J* = 13.6 Hz, H-17*β*), 3.38 (2H, s, H-18), 1.47 (3H, s, H-19), 1.94 (3H, s, H-20), 3.46 (2H, s, H-22), 3.77 (3H, s, H-24); B, ^13^C-DEPT NMR (CDCl_3_, 125 MHz) *δ*_C_: 214.87 (C-1), 76.36 (C-2), 42.64 (C-3), 171.70 (C-4), 194.92 (C-5), 134.50 (C-6), 149.38 (C-7), 42.12 (C-8), 44.33 (C-9), 136.62 (C-10), 71.51 (C-11), 34.46 (C-12), 26.94 (C-13), 46.74 (C-14), 145.48 (C-15), 115.09 (C-16), 66.76 (C-17), 50.11 (C-18), 25.87 (C-19), 20.56 (C-20), 166.03 (C-21), 41.27 (C-22), 166.71 (C-23), 52.62(C-24).


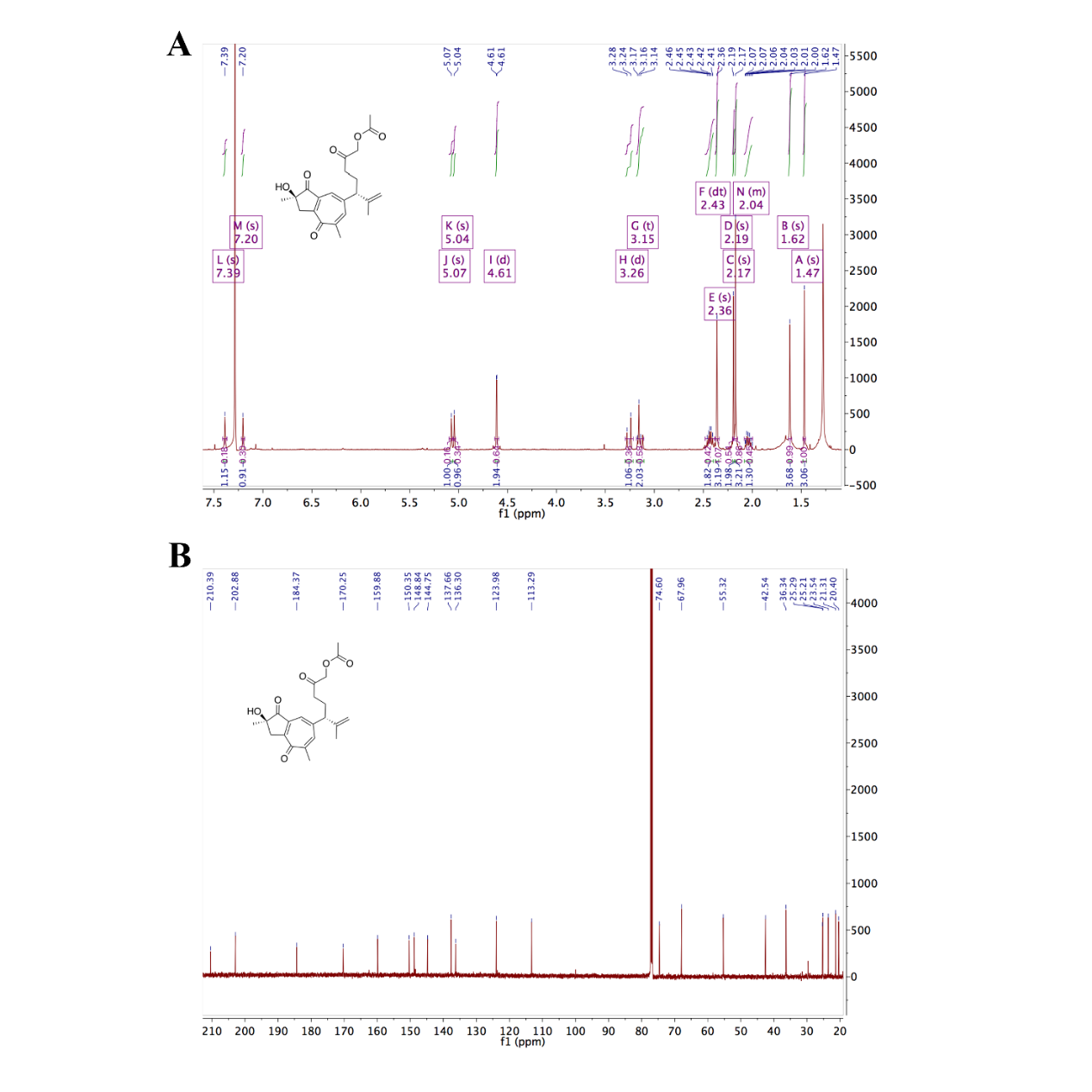
**Supplementary Figure 10. Structural information of derivative C-10.** ESI-MS m/z 409.16 [M + Na]^+^. A, ^1^H-NMR (CDCl_3_, 500 MHz) *δ* : 3.14 (1H, m, H-3*α*), 3.26 (1H, d, *J* = 19.8 Hz, H-3*β*), 7.38 (1H, s, H-7), 7.20 (1H, s, H-9), 3.14 (1H, m, H-11), 2.20 (1H, m, H-12*α*), 2.03 (1H, m, H-12*β*), 2.43 (2H, m, H-13), 4.61 (2H, d, *J* = 1.8 Hz, H-15), 5.05 (2H, d, *J* = 13.5 Hz, H-17), 1.61 (3H, s, H-18), 1.47 (3H, s, H-19), 2.35 (3H, s, H-20), 2.17 (3H, s, H-22); B, ^13^C-DEPT NMR (CDCl_3_, 125 MHz) *δ*_C_: 210.39 (C-1), 74.60 (C-2), 42.54 (C-3), 136.30 (C-4), 184.37 (C-5), 150.35 (C-6), 137.66 (C-7), 148.84 (C-8), 123.98 (C-9), 159.88 (C-10), 55.32 (C-11), 25.29 (C-12), 36.34 (C-13), 202.88 (C-14), 67.96 (C-15), 144.75 (C-16), 113.29 (C-17), 21.31 (C-18), 25.21 (C-19), 23.54 (C-20), 170.25 (C-21), 20.40 (C-22).


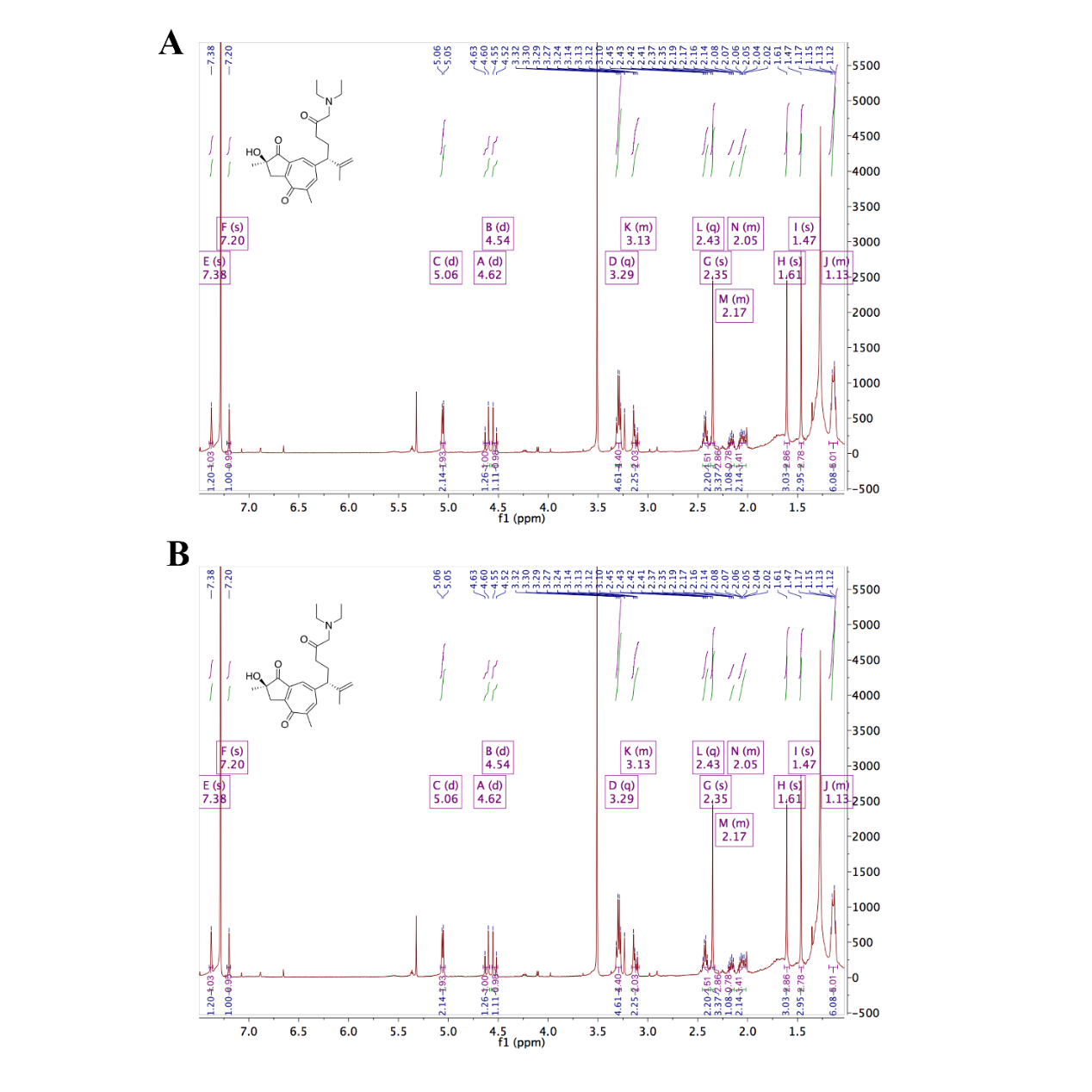
**Supplementary Figure 11. Structural information of derivative C-11.** ESI-MS m/z 422.23 [M + Na]^+^. A, ^1^H-NMR (CDCl_3_, 500 MHz) *δ* : 2.17 (1H, m, H-3*α*), 3.13 (1H, m, H-3*β*), 7.38 (1H, m, H-7), 7.20 (1H, m, H-9), 3.11 (1H, m, H-11), 2.05 (2H, m, H-12), 2.43 (2H, q, *J* = 7.1 Hz, H-13), 4.62 (1H, d, *J* = 16.9 Hz, H-15), 4.54 (1H, d, *J* = 17.0 Hz, H-15), 5.06 (2H, d, *J* = 6.5 Hz, H-17), 1.61 (3H, s, H-18), 1.47 (3H, s, H-19), 2.35 (3H, s, H-20), 3.29 (4H, q, *J* = 6.8 Hz, H-21,22), 1.14 (6H, dt, *J* = 11.5, 7.0 Hz, H-23,24); B, ^13^C-DEPT NMR (CDCl_3_, 125 MHz) *δ*_C_: 210.47 (C-1), 74.49 (C-2), 42.67 (C-3), 136.34 (C-4), 184.43 (C-5), 150.36 (C-6), 137.53 (C-7), 148.86 (C-8), 124.22 (C-9), 159.90 (C-10), 55.53 (C-11), 25.11 (C-12), 36.29 (C-13), 204.55 (C-14), 68.60 (C-15), 144.88 (C-16), 113.12 (C-17), 21.57 (C-18), 25.05 (C-19), 23.63 (C-20), 42.20 (C-21), 41.54 (C-22), 13.95 (C-23), 13.37 (C-24).


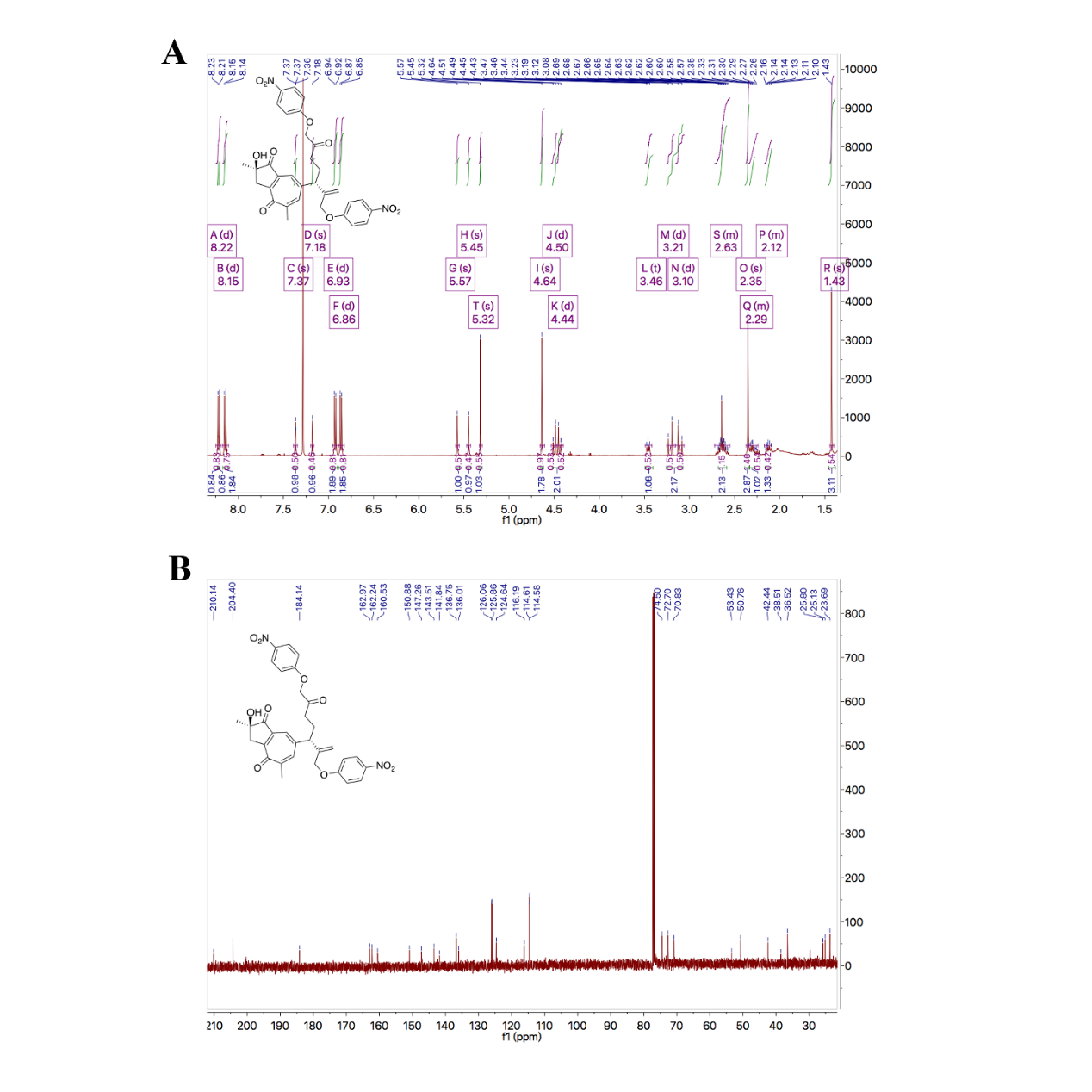
**Supplementary Figure 12. Structural information of derivative C-12.** ESI-MS m/z 625.18 [M + Na]^+^. A, ^1^H-NMR (CDCl_3_, 500 MHz) *δ* : 3.10 (1H, d, *J* = 20.1 Hz, H-3*α*), 3.21 (1H, d, *J* = 19.8 Hz, H-3*β*), 7.37 (1H, s, H-7), 7.18 (1H, s, H-9), 3.46 (1H, t, *J* = 7.6 Hz, H-11), 2.29 (1H, m, H-12), 2.12 (1H, m, H-12), 2.63 (2H, m, H-13), 4.64 (2H, s, H-15), 5.57 (1H, s, H-17*α*), 5.45 (1H, s, H-17*β*), 4.50 (1H, d, *J* = 12.5 Hz, H-18), 4.44 (1H, d, *J* = 12.5 Hz, H-18), 8.22 (2H, d, *J* = 9.2 Hz, H-2’,6’), 8.15 (2H, d, *J* = 9.2 Hz, H-2’’,6’’), 6.93 (2H, d, *J* = 9.2 Hz, H-3’,5’), 6.86 (2H, d, *J* = 9.2 Hz, H-3’’,5’’); B, ^13^C-DEPT NMR (CDCl_3_, 125 MHz) *δ*_C_: 210.14 (C-1), 74.50 (C-2), 42.44 (C-3), 136.01 (C-4), 184.14 (C-5), 150.88 (C-6), 136.75 (C-7), 147.26 (C-8), 124.64 (C-9), 160.53 (C-10), 50.76 (C-11), 25.80 (C-12), 36.52 (C-13), 204.40 (C-14), 72.70 (C-15), 143.51 (C-16), 116.19 (C-17), 70.83 (C-18), 25.13 (C-19), 23.69 (C-20), 162.24 (C-1’), 114.58 (C-2’,C-6’), 141.84 (C-4’), 125.86 (C-3’,C-5’), 162.97 (C-1’’), 114.61 (C-2’’,C-6’’), 141.84 (C-4’’),126.06 (C-3’’,C-5’’).


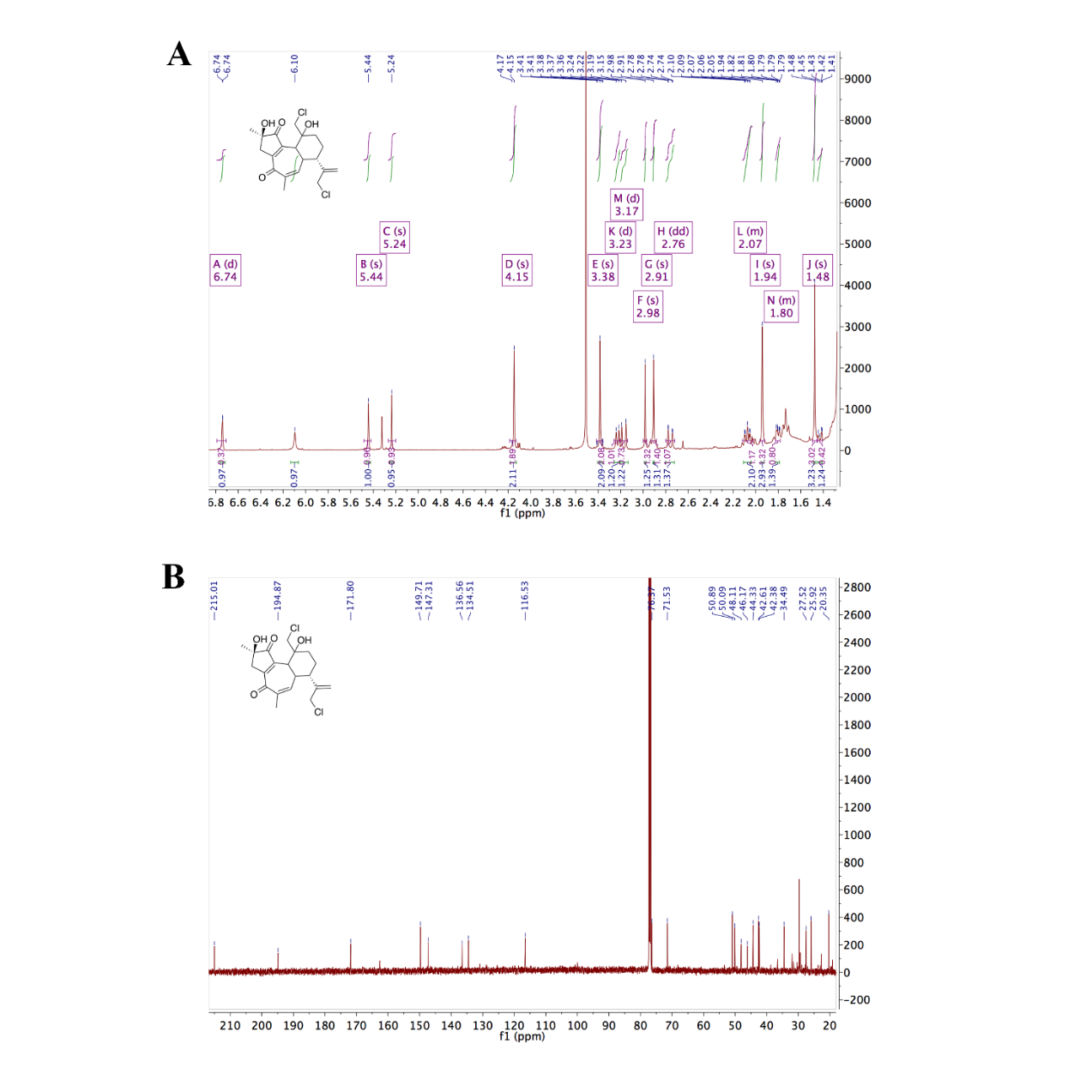
**Supplementary Figure 13. Structural information of derivative C-13.** ESI-MS m/z 403.13 [M + Na]^+^. A, ^1^H-NMR (CDCl_3_, 500 MHz) *δ* : 2.98 (1H, s, H-3), 2.91 (1H, s, H-3), 6.74 (1H, m, H-7), 3.20 (1H, m, H-8), 3.20 (1H, m, H-9), 2.07 (2H, m, H-12), 1.42 (1H, m, H-13*α*), 1.80 (1H, m, H-13*β*), 2.76 (1H, dd, *J* = 18.9, 2.2 Hz, H-14), 5.44 (1H, s, H-16*α*), 5.24 (1H, s, H-16*β*), 4.15 (2H, s, H-17), 3.38 (2H, s, H-18), 1.48 (3H, s, H-19), 1.94 (3H, s, H-20). ^13^C-DEPT NMR (CDCl_3_, 125 MHz) *δ*_C_: 215.01 (C-1), 76.37 (C-2), 42.61 (C-3), 171.80 (C-4), 194.87 (C-5), 134.51 (C-6), 149.71 (C-7), 42.38 (C-8), 44.33 (C-9), 136.56 (C-10), 71.53 (C-11), 34.49 (C-12), 27.52 (C-13), 46.17 (C-14), 147.31 (C-15), 116.53 (C-16), 48.11 (C-17), 50.09 (C-18), 25.92 (C-19), 20.35 (C-20).


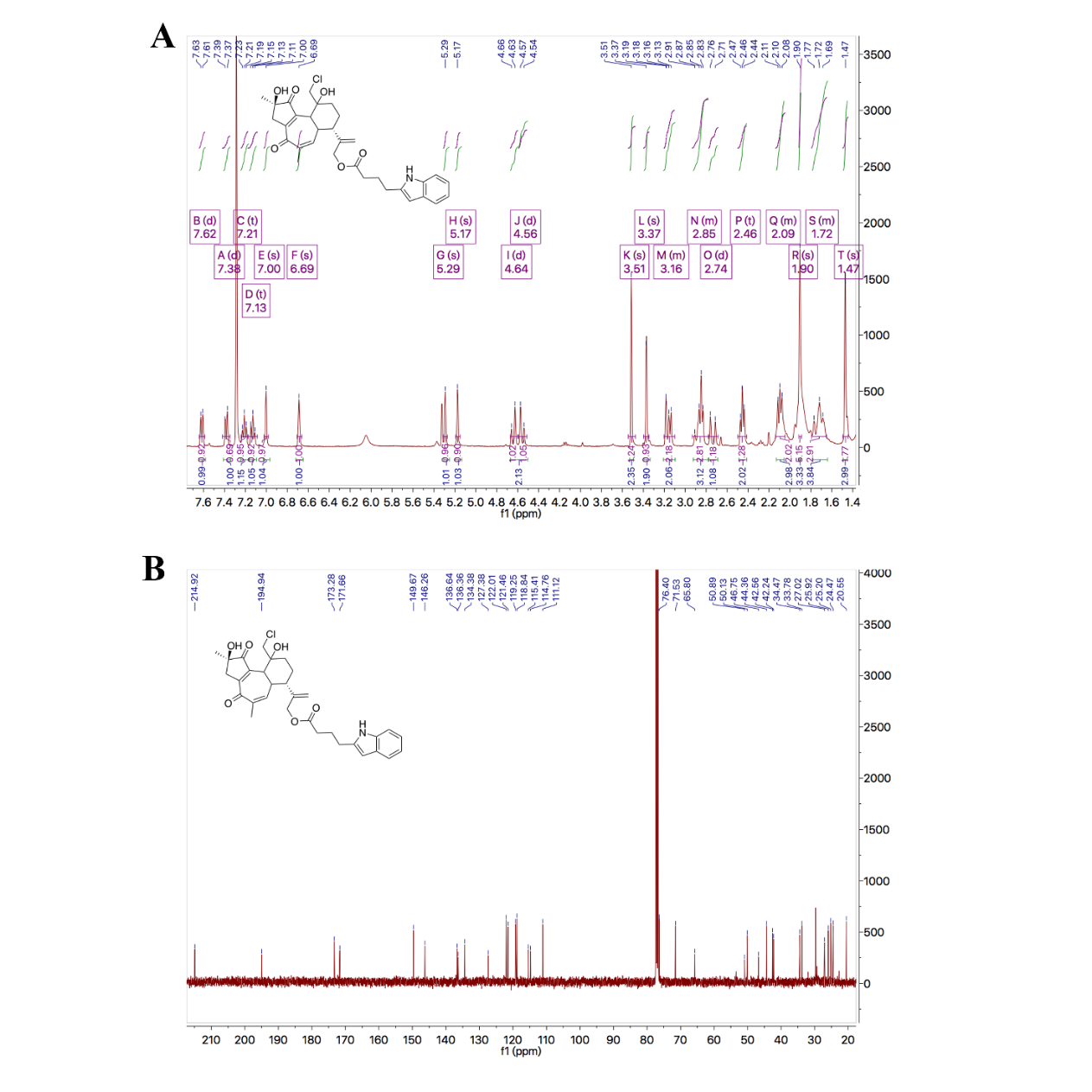
**Supplementary Figure 14. Structural information of derivative C-14.** ESI-MS m/z 588.21 [M + Na]^+^. A, ^1^H-NMR (CDCl_3_, 400 MHz) *δ* : 2.74 (1H, d, *J* = 18.9 Hz, H-3*α*), 3.16 (1H, m, H-3*β*), 6.69 (1H, s, H-7), 2.85 (2H, m, H-8), 3.16 (1H, m, H-9), 2.09 (2H, m, H-12), 1.70 (2H, m, H-13), 2.09 (1H, m, H-14), 5.29 (1H, s, H-16*α*), 5.17 (1H, s, H-16*β*), 4.64 (1H, d, *J* = 13.6 Hz, H-17*α*), 4.56 (1H, d, *J* = 13.5 Hz, H-17*β*), 3.37 (2H, s, H-18), 1.47 (3H, s, H-19), 1.90 (3H, t, *J* = 1.6 Hz, H-20), 2.85 (2H, m, H-22), 1.70 (2H, m, H-23), 2.46 (2H, t, *J* = 7.0 Hz, H-24), 7.00 (1H, s, H-26), 7.62 (1H, d, *J* = 7.9 Hz, H-29), 7.13 (1H, t, *J* = 8.0 Hz, H-30), 7.21 (1H, t, *J* = 7.7 Hz, H-31), 7.38 (1H, d, *J* = 9.2 Hz, H-32); B, ^13^C-DEPT NMR (CDCl_3_, 100 MHz) *δ*_C_: 214.92 (C-1), 76.40 (C-2), 42.56 (C-3), 171.66 (C-4), 194.94 (C-5), 134.38 (C-6), 149.67 (C-7), 42.24 (C-8), 44.36 (C-9), 136.64 (C-10), 71.53 (C-11), 34.47 (C-12), 27.02 (C-13), 46.75 (C-14), 146.26 (C-15), 114.76 (C-16), 65.80 (C-17), 50.13 (C-18), 25.92 (C-19), 20.55 (C-20), 173.28 (C-21), 33.78 (C-22), 24.47 (C-23), 25.20 (C-24), 115.41 (C-25), 122.01 (C-26), 136.36 (C-27), 127.38 (C-28), 118.84 (C-29), 119.25 (C-30), 121.46 (C-31), 111.12 (C-32).


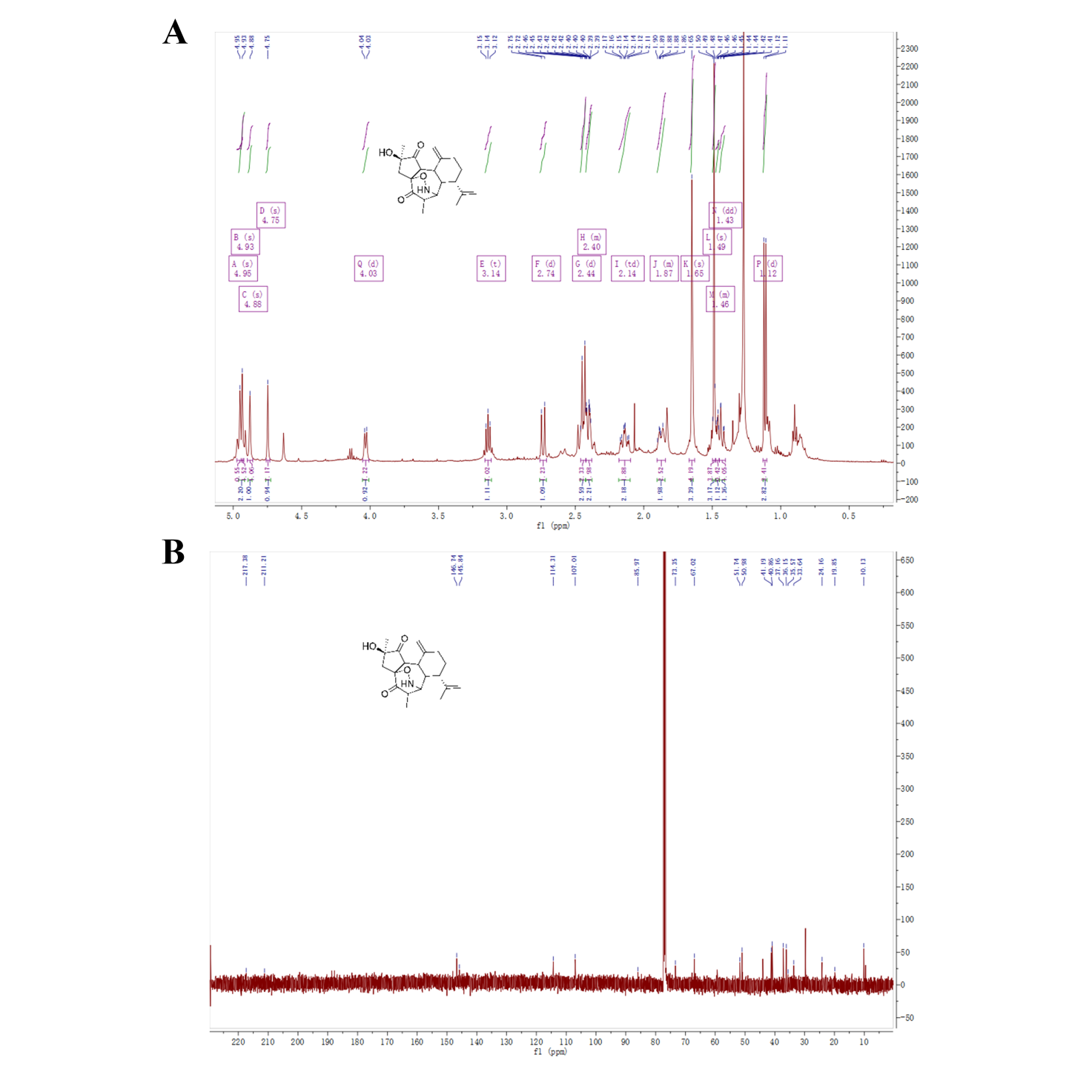
**Supplementary Figure 15. Structural information of derivative C-15.** ESI-MS m/z 368.18 [M + Na]^+^. ^1^H-NMR (CDCl_3_, 500 MHz) δ : 2.44 (1H, d, J = 10.7 Hz, H-3), 3.14 (1H, t, J = 7.4 Hz, H-6), 4.03 (1H, d, J = 7.6 Hz, H-7), 1.43 (1H, dd, J = 11.2, 1.6 Hz, H-8), 2.40 (1H, m, H-9), 2.74 (1H, d, J = 12.0 Hz, H-10), 2.14 (2H, td, J = 13.2, 4.8 Hz, H-12), 1.46 (1H, m, H-13α), 1.87 (1H, m, H-13β), 2.40 (1H,m, H-14), 4.95 (1H, s, H-16α), 4.88 (1H, s, H-16β), 1.65 (3H, s, H-17), 4.93 (1H, d, J = 11.6 Hz, H-18α), 4.75 (1H, d, J = 11.4 Hz, H-18β), 1.49 (3H, s, H-19), 1.12 (3H, d, J = 7.3 Hz, H-20). ^13^C-DEPT NMR (CDCl_3_, 125 MHz) δC: 217.38 (C-1), 85.97 (C-2), 36.15 (C-3), 73.35 (C-4), 217.38 (C-5), 40.86 (C-6), 67.02 (C-7), 44.02 (C-8), 41.19 (C-9), 50.98 (C-10), 107.01 (C-11), 37.16 (C-12), 33.64 (C-13), 51.74 (C-14), 145.84 (C-15), 114.31 (C-16), 19.85 (C-17), 146.74 (C-18), 24.16 (C-19), 10.13 (C-20).


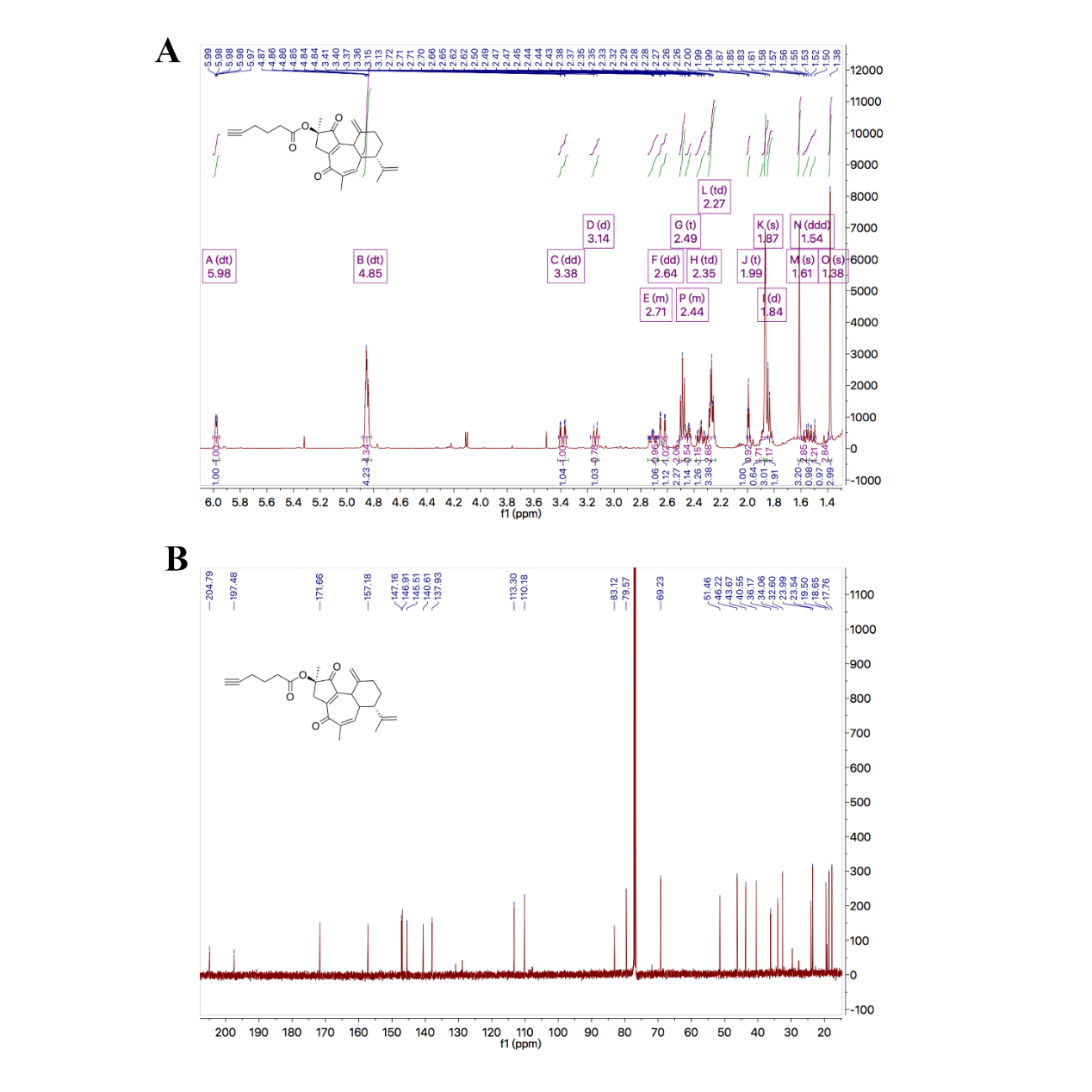
**Supplementary Figure 16. Structural information of positive probe.** ESI-MS m/z 429.20 [M + Na]^+^. ^1^H-NMR (CDCl_3_, 500 MHz) δ : 2.64 (1H, dd, J = 17.2, 2.4 Hz, H-3α), 3.38 (1H, dd, J = 17.3, 3.4 Hz, H-3β), 5.98 (1H, m, H-7), 2.71 (1H, tdd, J = 11.8, 4.9, 1.9 Hz, H-8), 3.14 (1H, d, J = 12.2 Hz, H-9), 2.44 (1H, m, H-12α), 2.27 (1H, m, H-12β), 1.54 (2H, m, H-13), 2.35 (1H, td, J = 11.9, 4.5 Hz, H-14), 4.85 (2H, m, H-16), 1.61 (3H, s, H-17), 4.85 (2H, m, H-18), 1.38 (3H, s, H-19), 1.87 (3H, s, H-20), 2.27 (2H, m, H-2’), 1.84 (2H, d, J = 7.1 Hz, H-3’), 2.49 (2H, t, J = 7.4 Hz, H-4’), 1.99 (3H, t, J = 2.6 Hz, H-6’). ^13^C-DEPT NMR (CDCl_3_, 125 MHz) δC: 204.79 (C-1), 79.57 (C-2), 40.55 (C-3), 157.18 (C-4), 197.48 (C-5), 140.61 (C-6), 137.93 (C-7), 43.67 (C-8), 46.22 (C-9), 145.51 (C-10), 147.16 (C-11), 34.06 (C-12), 36.17 (C-13), 51.46 (C-14), 146.91 (C-15), 113.30 (C-16), 18.65 (C-17), 110.18 (C-18), 23.99 (C-19), 19.50 (C-20), 171.66 (C-1’), 32.60 (C-2’), 23.54 (C-3’), 17.76 (C-4’), 83.12 (C-5’), 69.23 (C-6’).


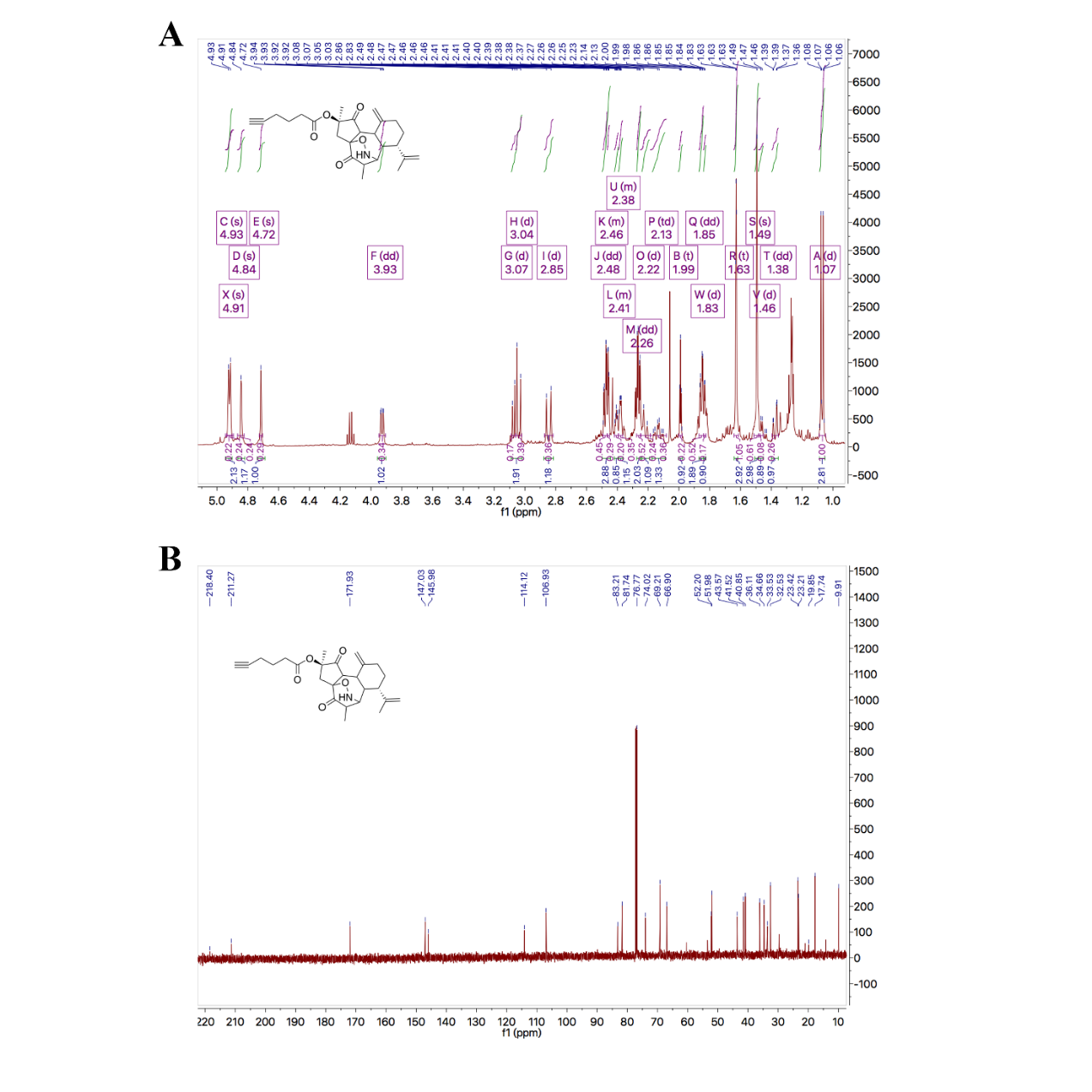
**Supplementary Figure 17. Structural information of negative probe.** ESI-MS m/z 429.20 [M + Na]^+^. A, ^1^H-NMR (CDCl_3_, 500 MHz) *δ* : 2.46 (1H, m, H-3*α*), 2.85 (1H, d, *J* = 14.7 Hz, H-3*β*), 3.93 (1H, dd, *J* = 7.5, 1.9 Hz, H-7), 1.38 (1H, dd, *J* = 11.3, 1.9 Hz, H-8), 2.22 (1H, d, *J* = 11.6 Hz, H-9), 3.04 (1H, d, *J* = 11.6 Hz, H-10), 2.41 (1H, m, H-12*α*), 2.13 (1H, td, *J* = 13.2, 4.9 Hz, H-12*β*), 1.83 (1H, m, H-13), 1.46 (1H, m, H-13), 2.38 (1H, m, H-14), 4.93 (1H, s, H-16), 4.84 (1H, s, H-16), 1.63 (3H, s, H-17), 4.91 (1H, s, H-18), 4.72 (1H, s, H-18), 1.49 (3H, s, H-19), 1.07 (3H, d, *J* = 7.3 Hz, H-20), 2.48 (2H, dd, *J* = 7.5, 2.1 Hz, H-2’), 1.85 (2H, dd, *J* = 7.2, 1.9 Hz, H-3’), 2.26 (2H, dd, *J* = 6.9, 2.6 Hz, H-4’), 1.99 (3H, t, *J* = 2.6 Hz, H-6’); B, ^13^C-DEPT NMR (CDCl_3_, 125 MHz) *δ*_C_: 211.27 (C-1), 81.74 (C-2), 34.66 (C-3), 74.02 (C-4), 218.40 (C-5), 40.85 (C-6), 66.90 (C-7), 43.57 (C-8), 41.52 (C-9), 51.98 (C-10), 106.93 (C-11), 36.11 (C-12), 33.53 (C-13), 52.20 (C-14), 145.98 (C-15), 114.12 (C-16), 19.85 (C-17), 147.03 (C-18), 23.21 (C-19), 9.91 (C-20), 171.93 (C-1’), 32.53 (C-2’), 23.42 (C-3’), 17.74 (C-4’), 83.21 (C-5’), 69.21 (C-6’).

**Supplementary Figure 18.
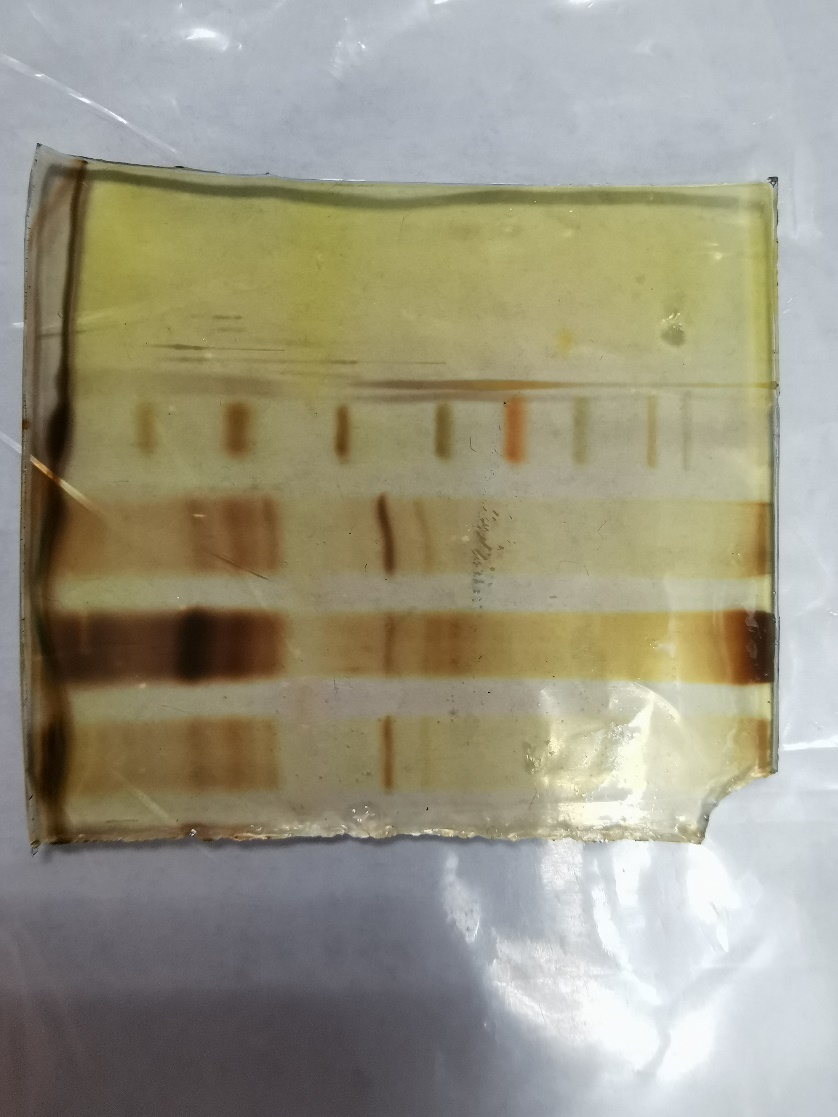

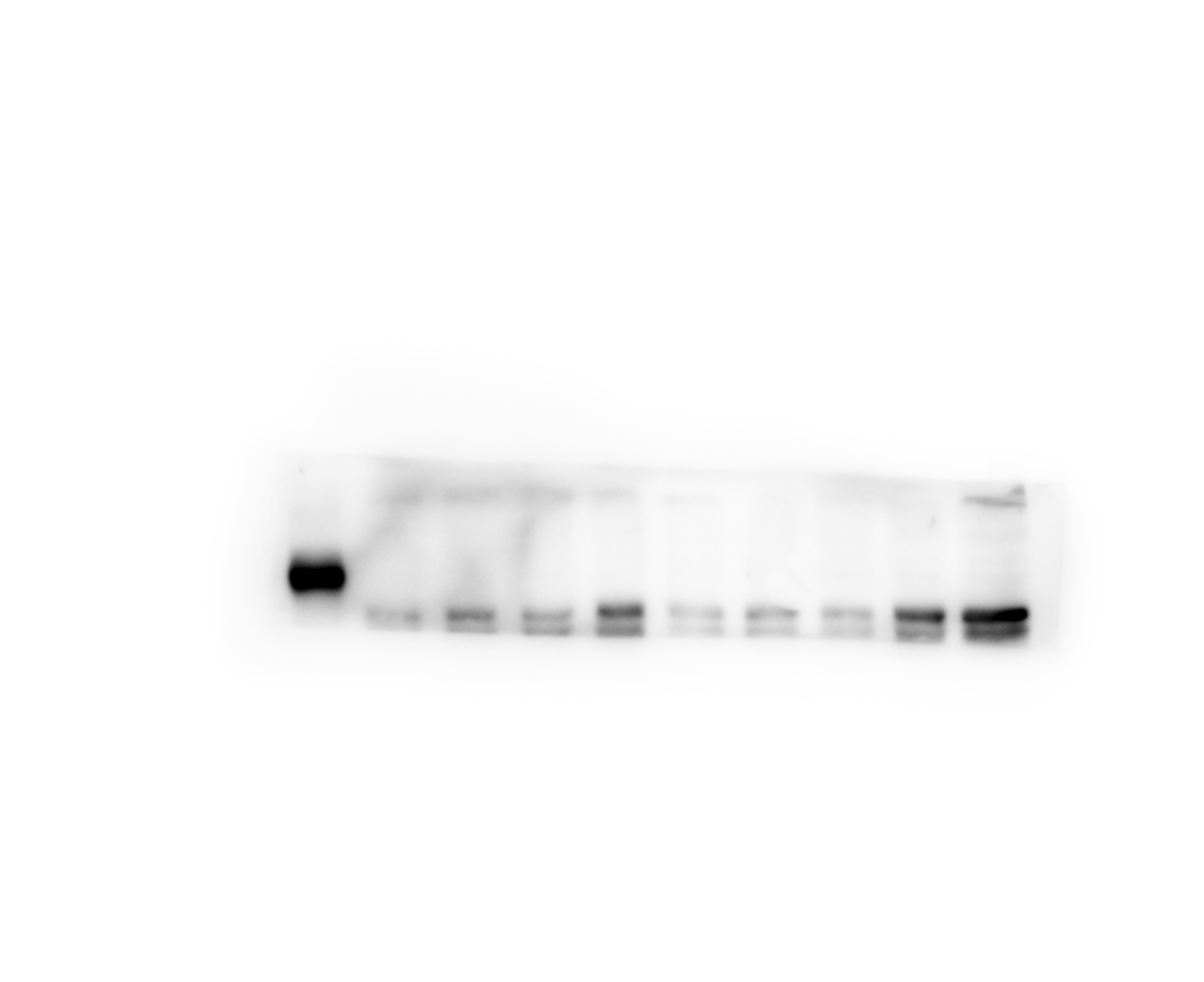
** Original results of SDS-PAGE Gel silver staining

**Supplementary

Figure 19.** Original western blot of PCBP2 in PC-3 cells under different concentrations of Curcusone C.

**Supplementary Figure 20.** Original western blot of GAPDH.

**Supplementary
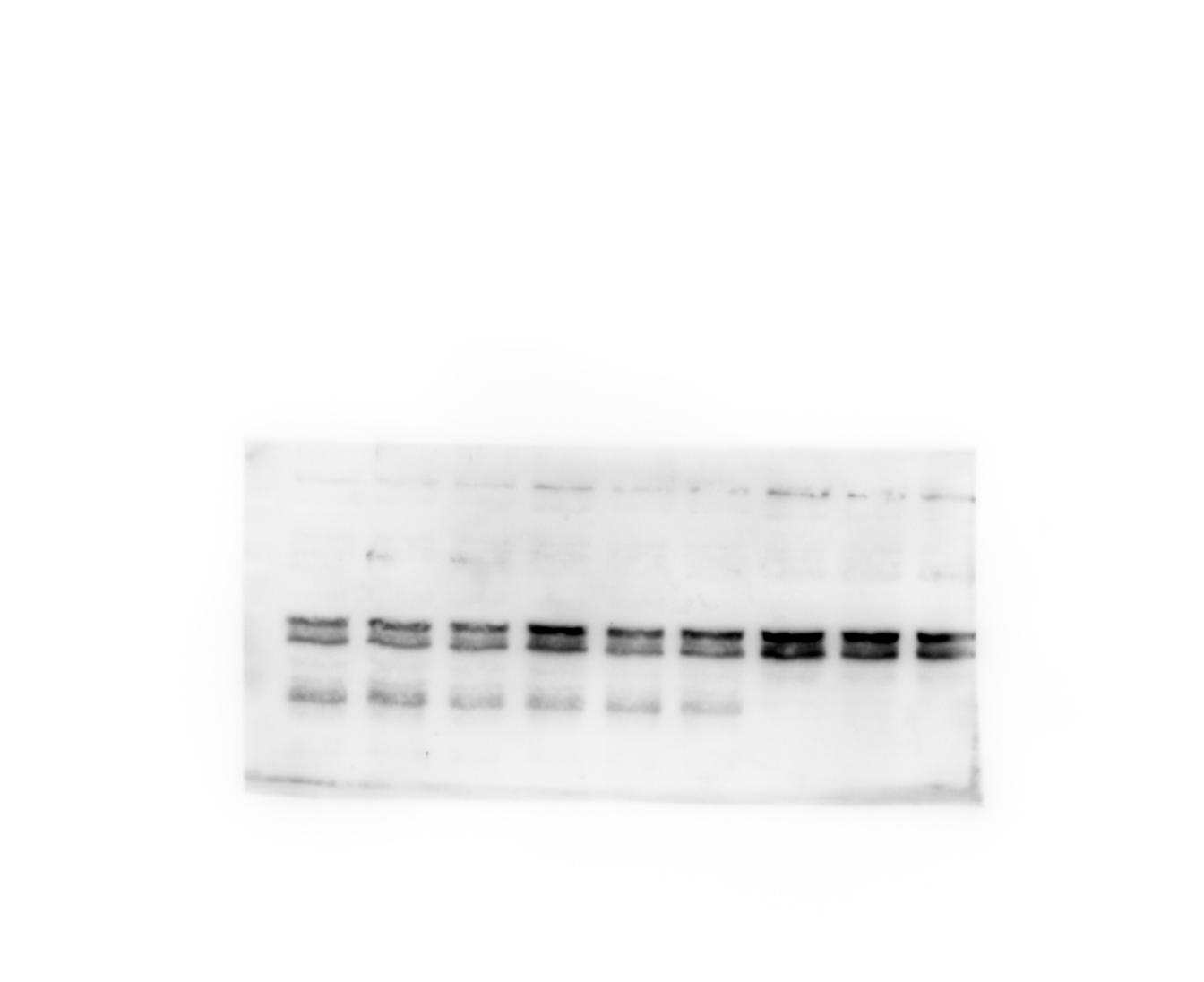

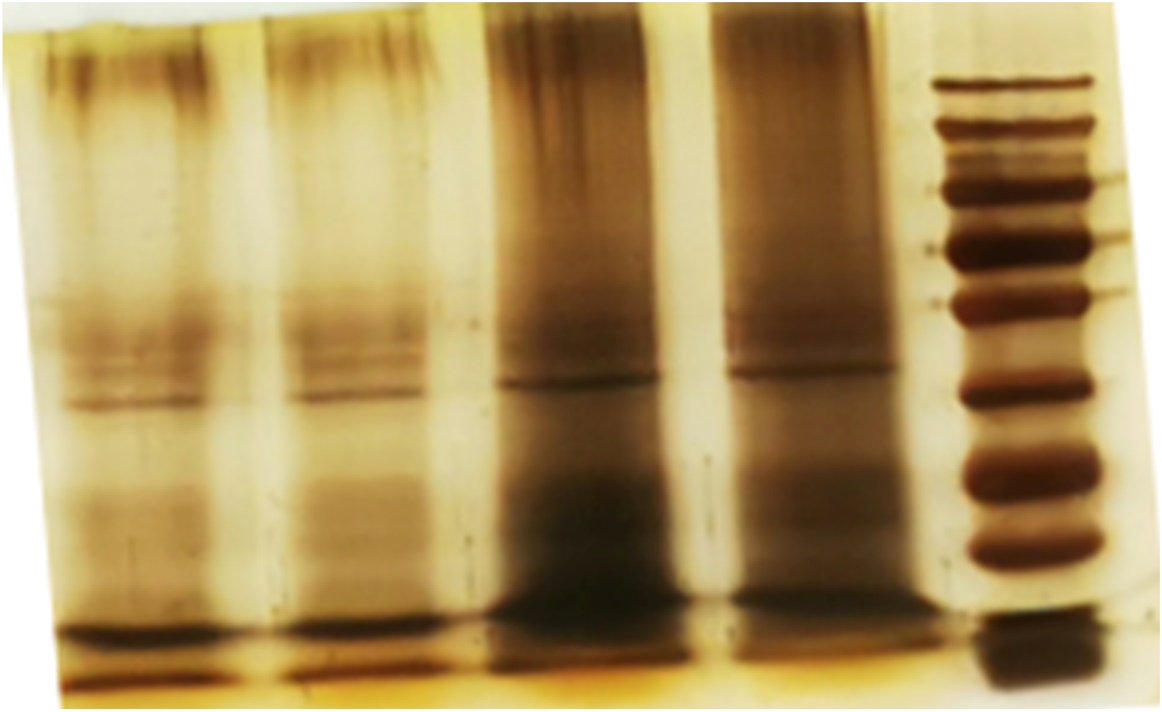
Figure 21.** Original result of competitive CC-ABPP silver staining.

**Supplementary

Figure 22.** Original result of DARTS.

**Supplementary

Figure 23.** Original western blot of FHL3.

**Supplementary Figure 24.** Original western blot of TGF-β1.

**Supplementary
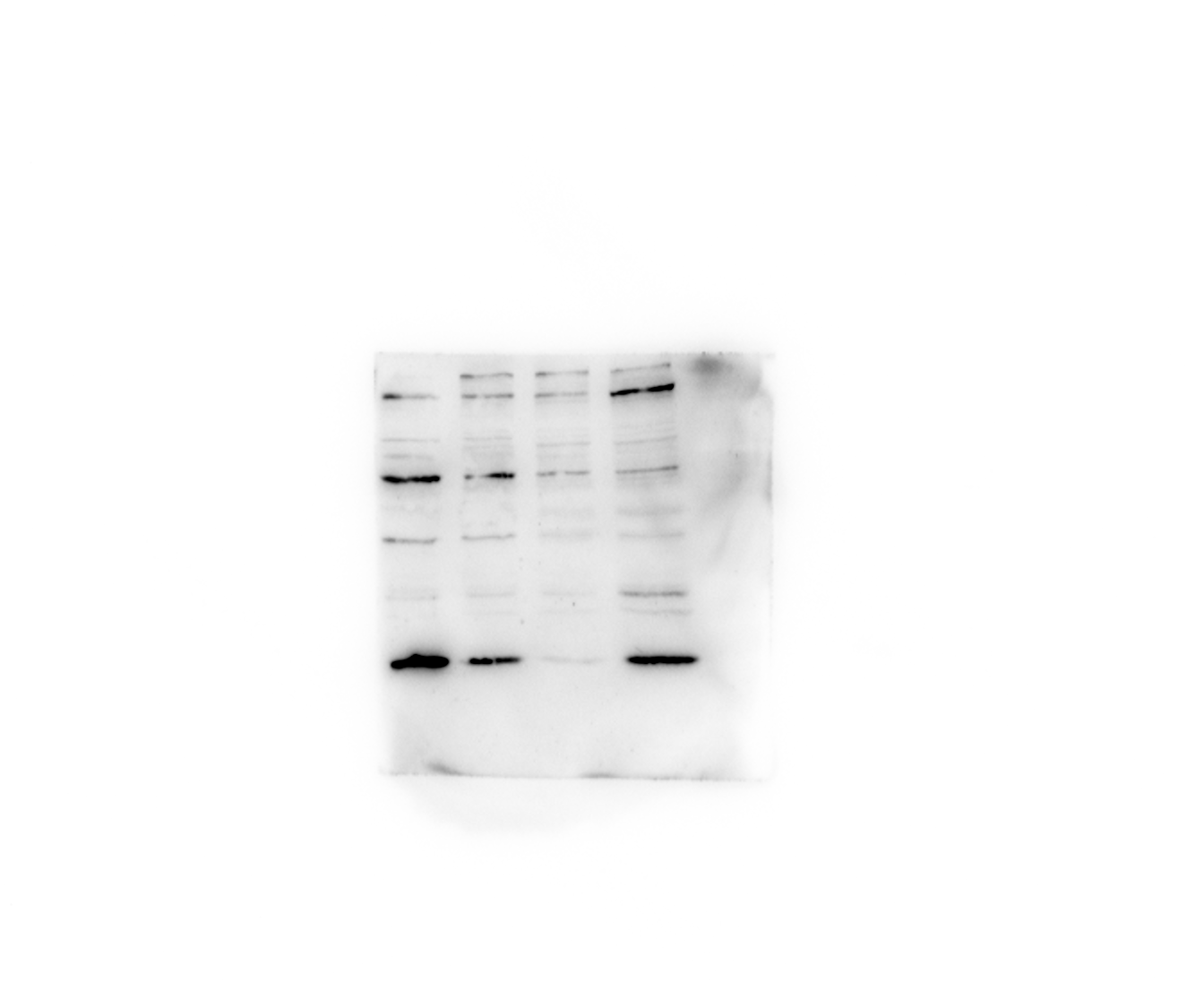
Figure 25.** Original western blot of Smad
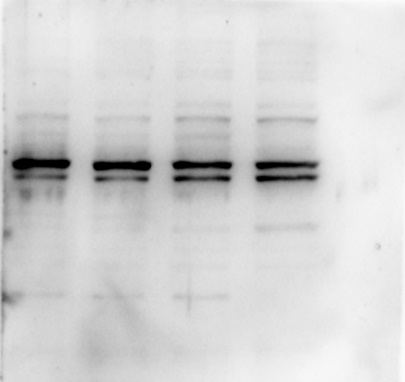
2.

**Supplementary

Figure 26.** Original western blot of p-Smad2.

**Supplementary

Figure 27.** Original western blot of Smad7.

**Supplementary

Figure 28.** Original western blot of p-Smad7.

**Supplementary Figure 29.** Original western blot of Bax.

**Supplementary
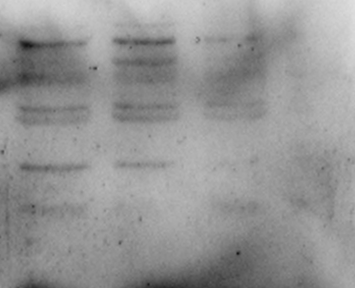


Figure 30.** Original western blot of Bcl-2.

**Supplementary

Figure 31.** Original western blot of Caspase3.

**Supplementary
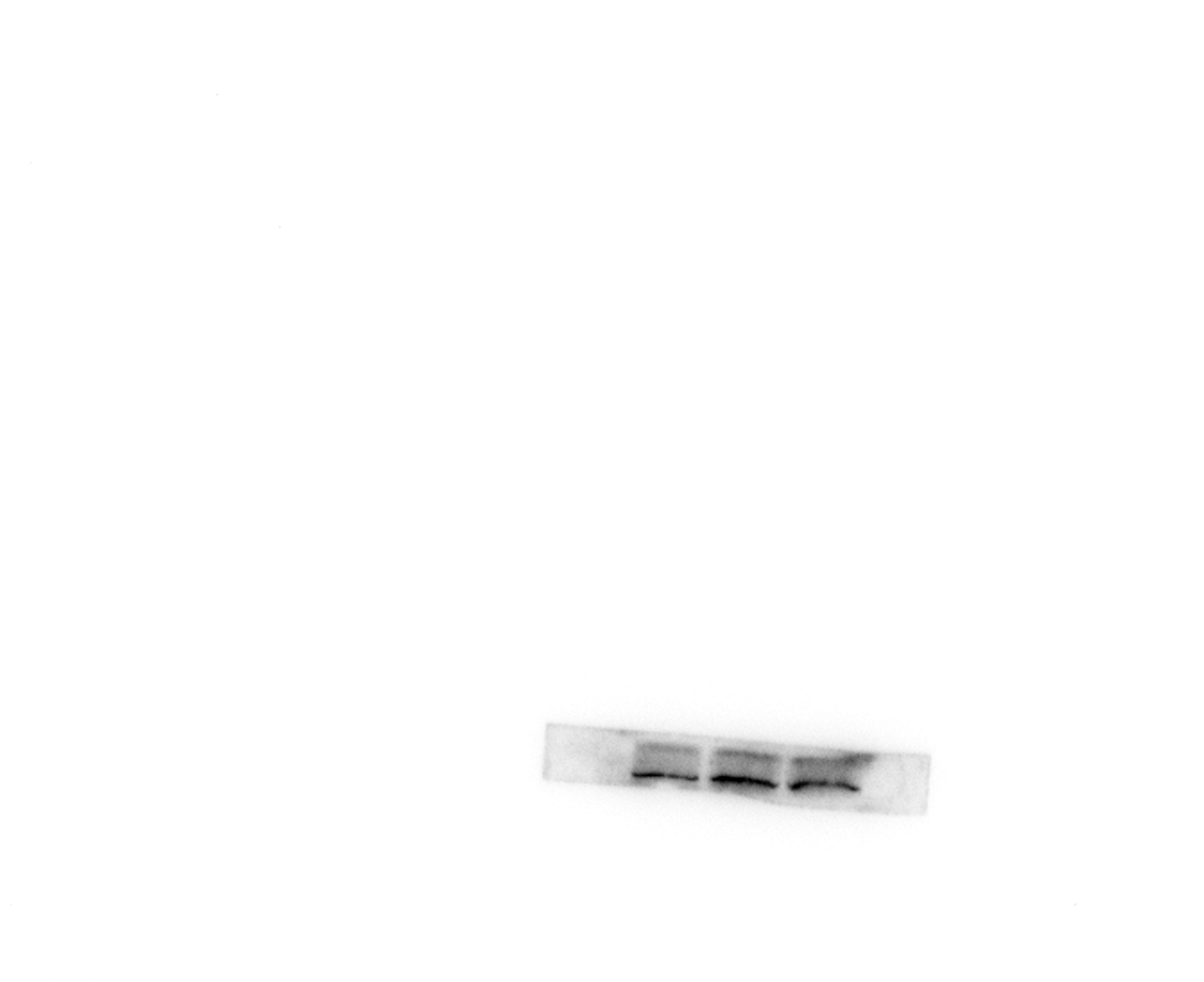
Figure 32.** Original western blot of Cleaved Caspase3.

**Supplementary

Figure 33.** Original western blot of PARP.

**Supplementary Figure 34.** Original western blot of Cleaved PARP.

**Supplementary Table 1 | The other nine proteins information in the top 10 protein between 25 and 35 kDa.**

| **Proteins** | **Locations** | **Functions** |
| --- | --- | --- |
| TACO1 | Mitochondria | Regulating the synthesis of mitochondrial synthetic cytochrome C oxidase |
| AK2 | Mitochondrial membrane | Regulating phosphotransferase composed of adenine nucleotides, energy homeostasis, oxidative stress and apoptosis in living organism |
| PRDX3 | Cytoplasm/Cell nucleus | Associating transcription factors, micrornas, tumor suppressors and response elements in tumor development and progression |
| SET | Cytoplasm/Cell nucleus | The interaction between SET and tumor suppressor PP2A can regulate protein stability or activation, regulating a variety of signaling pathways |
| PRDX4 | Cytoplasm | Mediating tumor cell epithelial-mesenchymal transformation (EMT) migration and tumorigenesis |
| SRPRB | Endoplasmic reticulun | Co-mediating NF-κB activation and regulation of apoptosis with SERP1 |
| NME1NME2 | - | NME1 is involved in the regulation of cancer metastasis, and NME2 is associated with histidine phosphotransferase action |
| PCNA | Cell nucleus | Coexisting with microchromosome maintenance (MCM) protein to participate in DNA replication and mediate cell division |
| PPA1 | Cytoplasm | Involving in breast cancer proliferation and metastasis by regulating Slug-mediated EMT |
